# Chalcones as Potential Ligands for the Treatment of Parkinson’s Disease

**DOI:** 10.3390/ph15070847

**Published:** 2022-07-10

**Authors:** Ewelina Królicka, Katarzyna Kieć-Kononowicz, Dorota Łażewska

**Affiliations:** Department of Technology and Biotechnology of Drugs, Faculty of Pharmacy, Jagiellonian University Medical College, Medyczna 9, 30-688 Kraków, Poland; ewelina.krolicka@uj.edu.pl (E.K.); mfkonono@cyf-kr.edu.pl (K.K.-K.)

**Keywords:** chalcones, Parkinson’s disease, dual target ligands, multitargeted ligands, MAO B inhibitors, COMT inhibitors, *α*-synuclein, adenosine receptor antagonists, neuroinflammation

## Abstract

Along with the increase in life expectancy, a significant increase of people suffering from neurodegenerative diseases (ND) has been noticed. The second most common ND, after Alzheimer’s disease, is Parkinson’s disease (PD), which manifests itself with a number of motor and non-motor symptoms that hinder the patient’s life. Current therapies can only alleviate those symptoms and slow down the progression of the disease, but not effectively cure it. So now, in addition to understanding the mechanism and causes of PD, it is also important to find a powerful way of treatment. It has been proved that in the etiology and course of PD, the essential roles are played by dopamine (DA) (an important neurotransmitter), enzymes regulating its level (e.g., COMT, MAO), and oxidative stress leading to neuroinflammation. Chalcones, due to their “simple” structure and valuable biological properties are considered as promising candidates for treatment of ND, also including PD. Here, we provide a comprehensive review of chalcones and related structures as potential new therapeutics for cure and prevention of PD. For this purpose, three databases (Pubmed, Scopus and Web of Science) were searched to collect articles published during the last 5 years (January 2018–February 2022). Chalcones have been described as promising enzyme inhibitors (MAO B, COMT, AChE), *α*-synuclein imaging probes, showing anti-neuroinflammatory activity (inhibition of iNOS or activation of Nrf2 signaling), as well as antagonists of adenosine A_1_ and/or A_2A_ receptors. This review focused on the structure–activity relationships of these compounds to determine how a particular substituent or its position in the chalcone ring(s) (ring A and/or B) affects biological activity.

## 1. Introduction

Parkinson’s disease (PD) is one of the most common neurodegenerative disorders (ND). Although its causes remain unknown, many researchers believe that the disease results from an interaction between genetic (e.g., gene mutation, age) and environmental (e.g., head trauma, pesticides) factors that lead to progressive neuronal degeneration in susceptible areas of the brain [1,2]. It affects 2–3% of the population aged ≥ 65 years and has an estimated incidence of 5 to >35 new cases per 100,000 people per year [3]. PD is associated with a reduction in the number of dopaminergic neurons in the *substantia nigra* (SN) and the presence of Lewy bodies (LB) and Lewy neurons (LN), both of which contain *α*-synuclein (*α*-syn) aggregates [4]. Damage to dopaminergic neurons reduces the release of dopamine (DA) into the striatum which leads to movement disorders such as bradykinesia, tremor, and rigidity. Clinical symptoms appear when nearly 60% of dopaminergic neurons are destroyed and the striatum DA concentration drops to about 20% [5]. PD is also related to non-motor symptoms (NMS), which could appear years before the diagnosis [5]. NMS are linked with changes in the neurotransmission systems (serotonergic, noradrenergic, or cholinergic). The most common NMS are depression and cognitive impairments, which vary among patients. Conducted studies showed that most patients with over 10 years of disease develop dementia [6]. The progress of the disease leads to severe damage to the elements of the autonomic, limbic, and somatic systems [7].

Braak et al. distinguished six stages of PD depending on symptoms [7]:

- Presymptomatic stages (**stage 1–2**), in which the pathology of inclusion bodies is confined to the medulla oblongata/pontine. Diagnosis of the disease in these stages is very rare and mainly non-motor symptoms (e.g., loss of smell, depressive symptoms) are present;
- **Stages 3–4**, in which the black matter and other grey nuclei of the midbrain and forebrain become the focus of pathological changes and symptoms are exacerbated. The lesions in the nervous system no longer respond to administered medication;
- The final **stages 5–6**, in which the process enters the mature neocortex. The disease manifests itself in all clinical dimensions and the progression of motor difficulties is so marked that the patient ceases to be independent.

**Figure 1:**
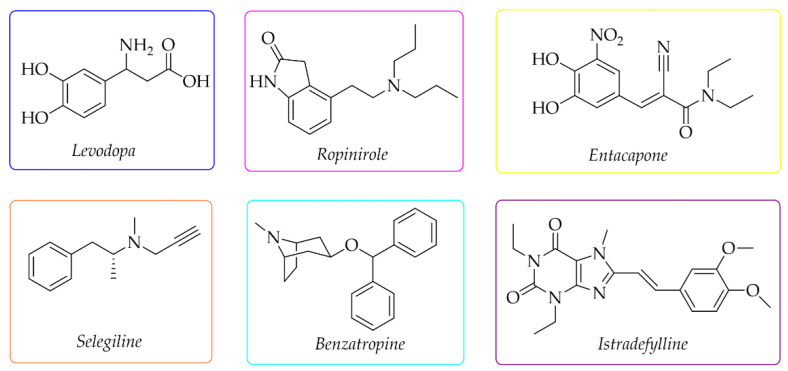
Chemical structures of the most commonly used drugs in Parkinson’s disease therapy and *istradefylline*.

Current therapy of PD can only alleviate symptoms and slow down the progression of the disease [1,2,3], but not cure it. The used drugs depend on the stage of PD. The first-choice drug is *levodopa* (Figure 1), a dopamine precursor which is easily transported to the brain and metabolised to DA. For early treatment, DA agonists are also used (e.g., *ropinirole*), as well as monoamine oxidase B (MAO B) inhibitors (e.g., *selegiline*), catechol-*O*-methyltransferase (COMT) inhibitors (e.g., *entacapone*), and drugs with cholinergic activity (e.g., *benzatropine*) (Figure 1) [8,9]. Recently, the first adenosine A_2A_ receptor antagonist—*istradefylline* (Japan-2013, FDA-2019)—was also approved as an add-on treatment to *levodopa* to increase its effectiveness and improve motor dysfunctions [10,11].

**Figure 2:**
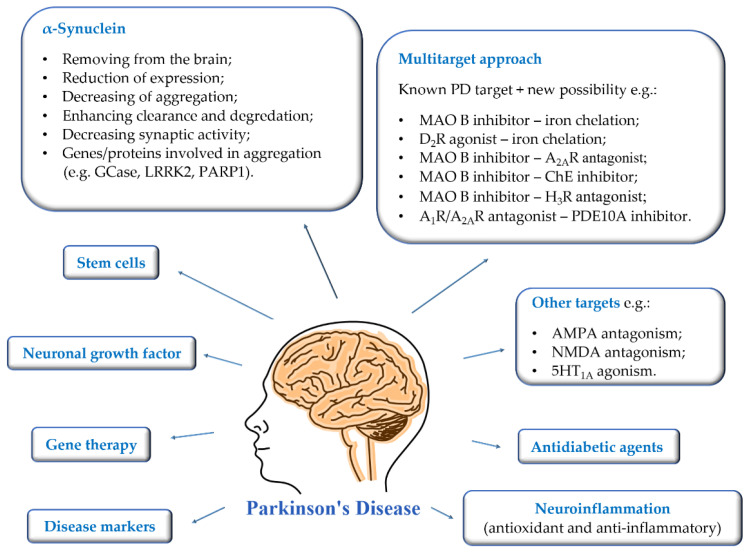
Current suggested approaches to the treatment, diagnosis, and prevention of Parkinson’s disease.

The growing number of PD patients necessitates the search for new strategies to combat this disease. The current research directions are shown in Figure 2.

First of all, the most important is to find disease markers that could be mass-used and allow early-stage detection. Then it will be possible to introduce treatment that could reduce the rate of disease progression. Gene therapy, intracerebral administration of neuronal growth factors, and stem cells are being developed at a significant pace. The mechanisms of brain stimulation are constantly improved, and new anatomical targets are sought to improve as many symptoms of the disease as possible. Moreover, new targets or multitarget approaches are proposed for therapy to prevent and reduce the rate of disease progression [3,12,13,14]. The utility of some of them is verified in clinical trials [1,3,13].

**Figure 3:**
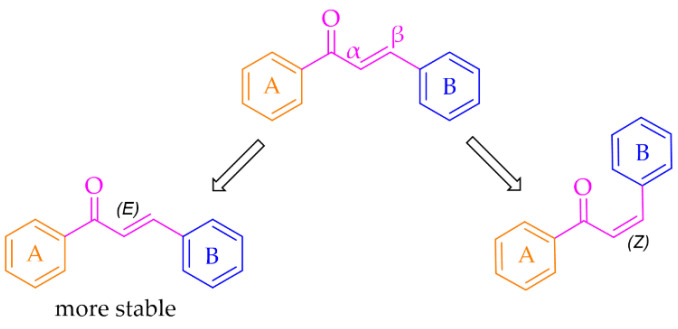
General structure of chalcones and possible isomers.

Especially interesting are multitarget strategies, as acting independently on a few targets involved in pathogenesis and development in PD could induce the desired pharmacological effect. In the search for new and effective drugs for PD, many scientists from universities and industry are engaged. Among many synthesised compounds, chalcones are promising candidates for this treatment due to a simple chemical scaffold occurring in many natural products. They can be found in vegetables, fruits, teas, and other plants [15]. They are open-chain flavonoids containing a benzylideneacetophenone scaffold in which two aromatic nuclei (ring **A** and **B**) are linked by an *α, β*-unsaturated carbonyl bond (Figure 3) [15,16]. Chalcones exhibit a lot of biological activities, e.g., anti-inflammatory, anti-bacterial, anti-cancer, anti-diabetic, anti-viral, anti-oxidant, as well as central nervous system (CNS) activities [17,18,19]. The presence of the *α, β*-unsaturated bond in the neighbourhood of the carbonyl group is responsible for the biological activity of these compounds. Elimination of this feature renders these compounds inactive [16]. Chalcones are Michael acceptors and could form covalent bonds with nucleophiles, e.g., the sulfhydryl group of cysteine residues in cellular peptides and proteins [20]. Moreover, chalcones can exist as two isomers *cis* (*Z*) and *trans* (*E*), but *E* isomers are more thermodynamically stable (due to the lack of steric interaction between the ring **B** and the carbonyl group) (Figure 3).

Furthermore, chalcones have small polar surface areas, which facilitates them to cross the blood–brain barrier (BBB) and act in CNS. This feature is mainly connected with the hydrophobic nature of the two aromatic nuclei of the **A** and **B** rings [21]. The most popular synthetic way of chalcones is of Claisen–Schmidt condensation. This method is experimentally simple and effective although sometimes time-consuming and yields by-products. Furthermore, chalcones are interesting structures as they can be precursors to the synthesis of 5- and 6-membered heterocycles by ring-closure reaction, e.g., derivatives of pyrazole, pyrrole, furan, or pyridine [22].

**Figure 4:**
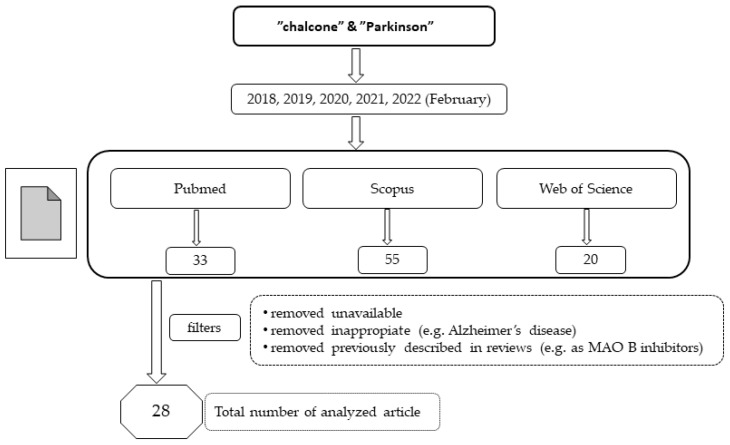
The way of selecting articles for this review.

In recent years, chalcones have attracted the attention of many researchers as new potential drugs for the treatment of PD due to the influence on many biochemical pathways, although the mechanism of these interactions is often unclear. A number of compounds were designed, synthesised, and pharmacologically evaluated. In this review, we present the results from studies conducted during the last five years (January 2018–February 2022). For this purpose, three databases (Pubmed, Scopus and Web of Science) were searched using the terms “chalcone” and “Parkinson”. The way of selecting articles for this review is presented in Figure 4.

**Figure 5:**
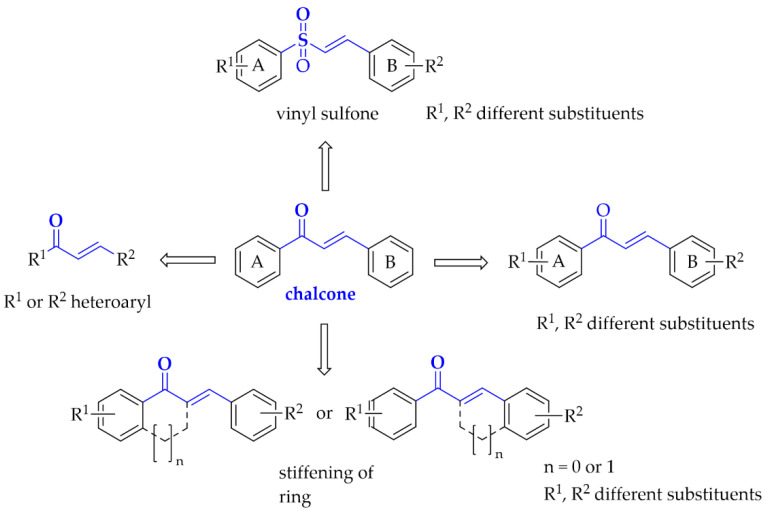
Structural modifications observed in chalcones with potential utility in Parkinson’s disease treatment.

Moreover, here we also describe compounds closely related to chalcones: aurones, benzoyl benzofurans, benzylidene-tetralones, benzylidene-indanones, and vinyl sulfones. General modification ways observed in chalcones connected with PD are shown in Figure 5. Compounds are discussed according to biological targets connected with PD: MAO B, *α*-synuclein, adenosine A_1_ and A_2A_, oxidative stress, and neuroinflammation.

## 2. Monoamine Oxidase B Inhibitors

**Figure 6:**
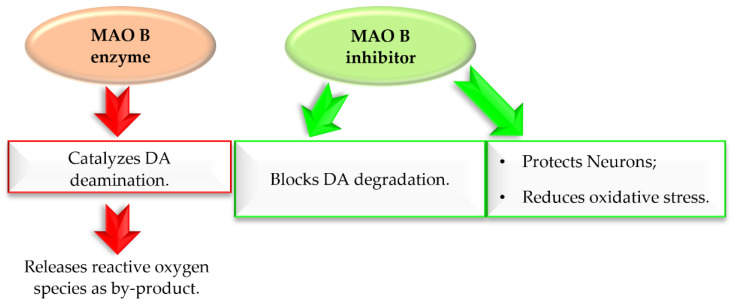
Effect of MAO B enzyme and MAO B inhibitor in Parkinson’s disease.

Monoamine oxidase B (MAO B) plays a key role in the pathogenesis of ND, including PD. It catalyses DA (in basal ganglia), which produces reactive oxygen species (ROS) (Figure 6) [23]. The increased level of ROS causes a decline in mitochondrial function and viability of neurons leading to neurodegeneration. MAO B enzyme is present especially in serotonergic and histaminergic neurons, as well as in glial cells, especially astrocytes, and, outside the nervous system, in platelets and lymphocytes [23]. With age, activity of MAO B in brain tissues increases. Since 40 years ago, three MAO B inhibitors (Figure 5) have been used in the treatment of PD, mostly in the combination with *levodopa*, which allows reducing the effective dose of this drug. Recent in vitro and in vivo studies showed that MAO B inhibition not only delays dopaminergic neurons degeneration in the brain, but also abolishes the formation and transmission of *α*-synuclein aggregates [24]. Thus, MAO B inhibition is an important target in the search for new drugs for PD.

During the last years, many different scaffolds were investigated as MAO B inhibitors [25], chalcones being among them. Recently, Guglielmi et al. [26] reviewed chalcones as MAO B inhibitors. The reader can find therein a comprehensive report about the structure–activity relationship of a number of chalcone derivatives showing different MAO B inhibitory activities. In this article, we focused on chalcones that were not described in the previous publication and analysed the effect of substituents on MAO B inhibition. Compounds were divided into three main groups: monosubstituted chalcones, disubstituted chalcones, and multitarget chalcones.

### 2.1. Monosubstituted Chalcones

**Figure 7:**
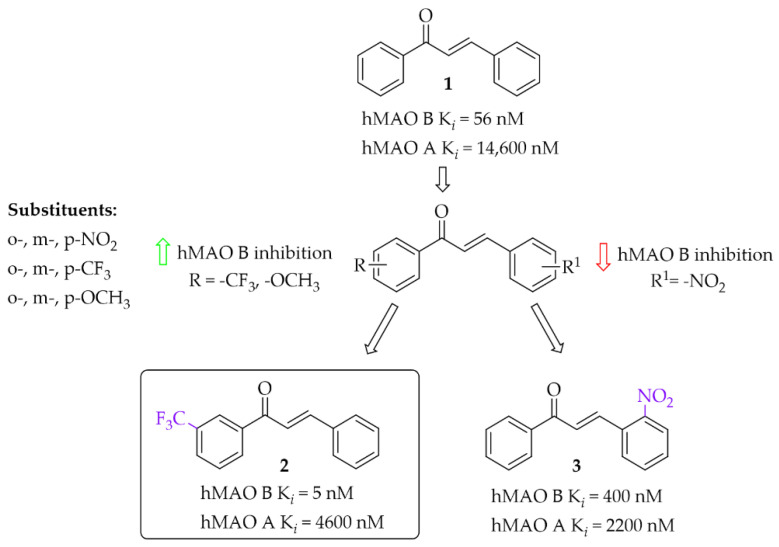
Mono-modification of chalcone ring—the effect of substituents on the inhibitory activity of human MAO B [27].

Iacovino et al. [27] described human MAO B (hMAO B) inhibitors as a series of mono-substituted chalcones containing electron-donating (-CF_3_, -CH_3_) or electron-withdrawing (-NO_2_) substituents. All of 17 synthesised compounds were first tested in vitro for cytotoxicity on cancer cell lines (ovarian carcinoma A2780, colorectal adenocarcinoma HT-29, and biphasic mesothelioma MSTO-211H). Then, compounds that were non-cytotoxic in all tested lines (GI_50_ > 20 µM) were further evaluated for inhibitory activity against hMAO B and human MAO A (five compounds). Due to the small number of compounds it was difficult to see any general relation between structure and activity but the strongest inhibitor was a compound containing a -CF_3_ group in the *meta* position (compound **2**; Figure 7) at the **A** ring. This compound acted as a competitive reversible inhibitor of hMAO B, showing an inhibition constant value with a K*_i_* of 5 nM. Interestingly, “pure” chalcone moiety (compound **1**; Figure 7) showed good inhibitory activity with a K*_i_* of 56 nM. Substitution in the **B** ring with -NO_2_ substituent especially in the *ortho* position (compound **3**; Figure 7) was unfavourable as it weakened the inhibitory activity (K*_i_* = 400 nM). All compounds proved to be weak hMAO A inhibitors (K*_i_* ≥ 2.2 μM). Molecular docking studies of **2** showed that this compound binds to the active site with the -CF_3_ group in the entrance to cavity space, whereas crystallographic analysis (crystal structure of hMAO B with compound **2**) showed that the -CF_3_ group played a key role in this orientation to the active site [27].

### 2.2. Disubstituted Chalcones

#### 2.2.1. Chalcones Containing a Thioether Group

**Figure 8:**
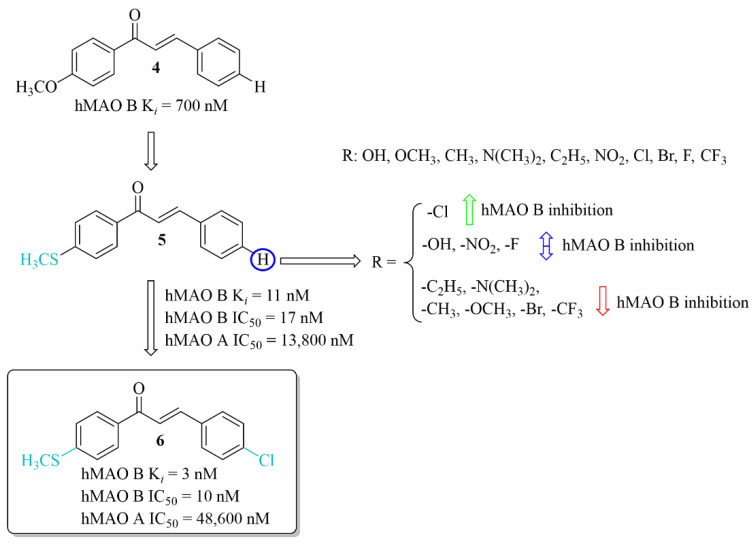
Thioethers and the most potent compound in the series [28].

The development of new potent hMAO B inhibitors with a thioether group was led by Mathew et al. [28]. The researchers synthesised eleven chalcones and evaluated their ability to inhibit hMAO B and hMAO A. The thiomethoxy group (-SCH_3_) was introduced into the **A** ring at the *para* position, and the various substituents at the *para* position in the **B** ring. All compounds exhibited good inhibitory activity for hMAO B with IC_50_ values < 150 nM. The parent compound **5** (without any substituent on the **B** ring) (Figure 8) showed good inhibition of hMAO B (IC_50_ = 17 nM; K*_i_* = 11 nM). The obtained results were better than for the methoxylated analogue **4** (K*_i_* = 700 nM). Thus, the -SCH_3_ group at the *para* position of the chalcone **A** ring potentiated the inhibitory effect of hMAO B. Substituents in the **B** ring had a different influence on inhibitory activity. The introduction of -N(CH_3_)_2_, -CH_2_CH_3_ and -CH_3_ groups as well as -OCH_3_, -CF_3_ and -Br to this ring resulted in a decrease of inhibitory activity in comparison with compound **5**. Compounds with -OH, -NO_2_ or -F group had comparable inhibitory activity to the compound **5**. Only compound with the -Cl substituent on the **B**, compound **6** (Figure 8) showed slightly higher inhibitory activity (IC_50_ = 10 nM; K*_i_* = 3 nM) than compound **5**. Opposite to that all compounds had a weak inhibitory activity of hMAO A (IC_50_ > 5 µM). Both compounds **5** and **6** (as the most promising) were selected for further studies. Kinetic and reversibility studies confirmed competitive and reversible inhibition of hMAO B by them. Moreover, very high blood–brain barrier permeability for both compounds was estimated in PAMPA assay (Pe > 15 × 10^−6^ cm/s). Furthermore, little toxicity to Vero cells (IC_50_ > 110 μg/mL) and ability of compounds to lower the H_2_O_2_-induced ROS level in these cells was observed [28].

#### 2.2.2. Oxygenated Chalcones

**Figure 9:**
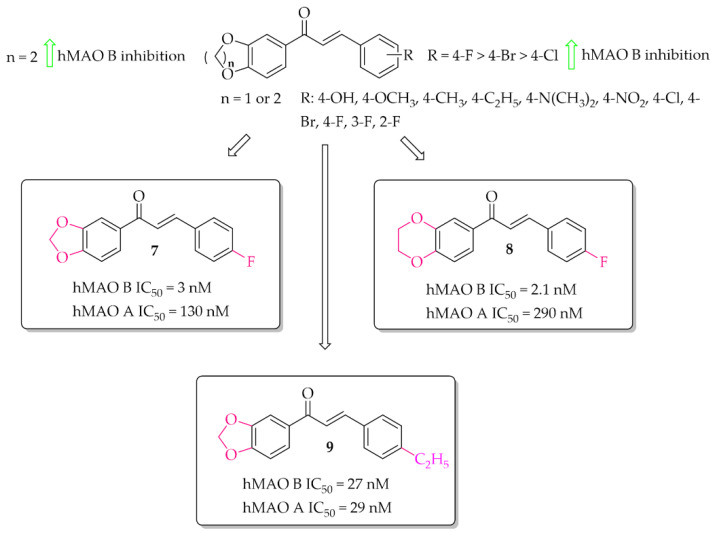
Oxidised chalcones with the most potent compound in the series [29].

Oxygen derivatives of chalcones as hMAO B inhibitors were investigated by Parambi et al. [29]. Researchers synthesised 26 compounds containing either methylenedioxymethyl (1,3-benzodioxole) or ethylenedioxymethyl (1,4-benzodioxane) group as the **A** ring of the chalcone moiety. Changes were made on the **B** ring of the chalcone moiety where different substituents were introduced in the *para* position. All tested compounds showed strong inhibitory activity against hMAO B (IC_50_ < 70 nM). Structure–activity relationship analysis showed that the derivatives containing benzo-1,4-dioxane ring exhibited better hMAO B inhibitory activity than the chalcones containing benzo-1,3-dioxole ring. On the other hand, in both cases a very positive effect of halogen substituents, particularly the fluorine atom, was observed. The most active compounds in these series showed very high hMAO B inhibition with an IC_50_ below 5 nM, i.e., compound **7** (IC_50_ = 3 nM; Figure 9) and compound **8** (IC_50_ = 2.1 nM; Figure 9). All compounds were also tested for hMAO A inhibitory activity and results showed that most compounds potently inhibited this enzyme, too. The strongest hMAO A inhibitor was compound **9** (Figure 9) with similar activity towards both enzymes (hMAO A IC_50_ = 29 nM; hMAO B IC_50_ = 27 nM). Kinetic and reversibility studies of the most potent compounds (**7**–**9**) showed competitive and reversible inhibition of hMAO B by compounds **7** and **8**, and the same kind of inhibition by compound **9** for hMAO A. In cytotoxicity studies in rat spleen cells, compounds **7** and **8** showed a very weak toxic effect at a dose of 200 μg/mL (11% and 4% cell death, respectively). These results encouraged researchers to check activity of these compounds **7** and **8** in in vivo studies [30]. Thus, compounds **7** and **8** were evaluated in the haloperidol-induced murine model of PD. Catalepsy was induced by haloperidol at the dose of 1 mg/kg/day p.o. Compounds were tested in three doses: 10, 20, and 30 mg/kg/day p.o. for 21 days. Two control groups were used. In one of the disease control groups (a standard group), animals were treated with *levodopa* (20 mg/kg/day p.o.) plus *carbidopa* (2 mg/kg/day p.o.). Results showed a significant reduction of catalepsy by these compounds especially at the dose of 30 mg/kg/day p.o. The locomotor and exploratory behaviour of animals were evaluated in the open field test. Both compounds showed positive effects comparable with the standard group. A further anti-anxiety effect was observed in the hole-board test also at the dose of 30 mg/kg/day p.o. Moreover, compounds were also tested in the narrow beam-walk test to evaluate their influence on motor coordination and balance, especially the hind limbs. The time taken by mice to traverse the beam was dose-dependently decreased by both compounds, but more by compound **8**. Further studies showed that these compounds increased the levels of anti-oxidant markers (SOD, CAT, and GSH) and decreased oxidative stress marker MDA. Additionally, compounds dose-dependently increased levels of neurotransmitters such as DA, acetylcholine, noradrenaline, and serotonin. A reduction of brain damage (neurofibrillary tangles and plaques) was also observed under a light microscope at 100× in the groups treated by both compounds with the dose of 30 mg/kg/day p.o. Thus, these results showed that the tested chalcones, especially **8**, are promising candidates for the treatment of PD [29,30].

**Figure 10:**
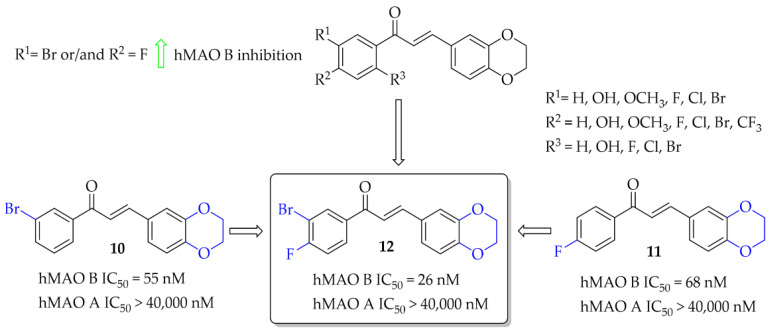
1,4-Benzodioxan-substituted chalcones and the most potent compounds in the series [31].

A series of 28 chalcone derivatives containing a 1,4-benzodioxane moiety was synthesised and evaluated for hMAO B and hMAO A inhibitory activity by Kong et al. [31]. Most of the compounds showed good inhibitory activity with IC_50_ < 350 nM. Structure–activity relationship analysis showed that the 1,4-benzodioxane moiety had a good influence on inhibitory activity, the same as a halogen atom, especially -Br (compound **10**; Figure 10) or -Cl at the 3 or 6 position of the ring **A**. Compound **11** (Figure 10) with 4-F substituent had similar affinity to compound **10** (**10**: IC_50_ = 55 nM; **11**: IC_50_ = 68 nM). The most potent in the whole series was compound **12** (Figure 10) with an IC_50_ value of 26 nM. None of compounds showed inhibition of hMAO A with IC_50_ < 40 μM. Performed kinetics and reversibility studies of compound **12** confirmed competitive and reversible inhibition of hMAO B. Cytotoxicity studies of compounds **10** and **12** were carried out in BV2 microglia cells and showed no significant decrease in cellular viability at tested concentrations (5 μM and 25 μM) [31].

#### 2.2.3. Chalcocoumarin Hybrids

**Figure 11:**
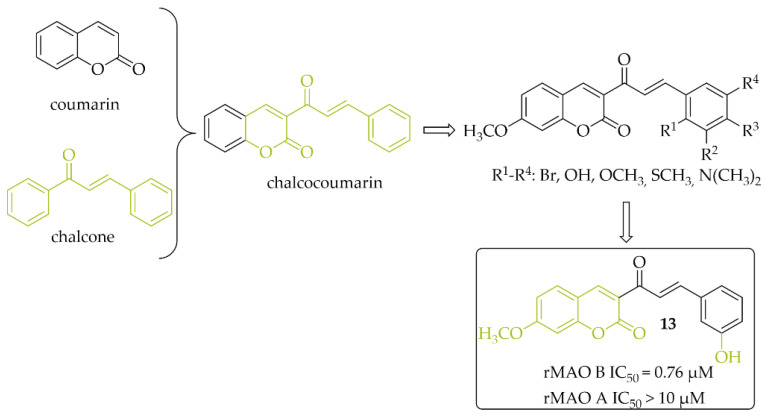
Chalcocoumarin hybrids and the most potent compound in the series [32].

A series of 14 chalcocoumarin hybrids were designed as potential hMAO B inhibitors by Moya-Alvarado et al. [32]. Tests were performed using rat brain mitochondria as source of MAO B (rMAO B) and MAO A (rMAO A). Several compounds showed affinity for rMAO B at low micro- and submicromolar concentrations. The most active compound **13** (Figure 11) had an IC_50_ value of 0.76 µM. None of the compounds showed inhibitory activity towards rMAO A (IC_50_ > 10 µM). Compound **13** also was examined by molecular modeling, ADMET prediction, docking, and MM/GBSA calculations. The results showed that compound **13** has a suitable interaction with the active site of rMAO B by matching the distance near the nitrogen atom of the polar nitrogen ring of the alloxazine FAD and forms an interaction that is not redundant to the inhibition of rMAO B. Thus compound **13** could be a good lead structure for further modification in the search for potent chalcocoumarin as MAO B inhibitors [32].

### 2.3. Dual and Multi-Target Monoamine Oxidase B Inhibitors

#### 2.3.1. Monoamine Oxidase B and Catechol-*O*-Methyltransferase Inhibitors—Chalcones with a Nitrocatechol Moiety

**Figure 12:**
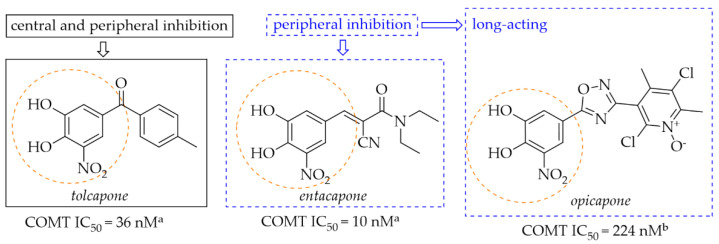
Chemical structures of second (*tolcapone* and *entacapone*)- and third (*opicapone*)-generation COMT inhibitors; ^a^IC_50_ values in rat liver homogenates, data from ref. [36], ^b^IC_50_ values in rat liver homogenates, data from ref. [38].

Catechol-*O*-methyltransferase (COMT) is expressed in non-neuronal tissues both in the brain and in the periphery. In the peripheral tissue it is in a soluble cytosolic form whereas in the brain it is in a membrane-bound form (MB-COMT) [33]. The COMT enzyme is a major catabolic regulator of catecholamine neurotransmitters (such as DA, noradrenaline, or epinephrine), hormones, and xenobiotics [33,34]. It participates in the catalytic transfer of the methyl group to these catechols which produces inactive metabolites. Especially MB-COMT is responsible for metabolism of DA. In a dopaminergic system, COMT also metabolises *levodopa*, thereby inhibiting DA synthesis. COMT inhibitors such as *entacapone*, *tolcapone* and *opicapone* are used in the therapy of PD (Figure 12). All of them possess a nitrocatechol moiety which is believed to be preferential in the inhibiting COMT enzyme [35]. Mostly, they are used as an add-on therapy to *levodopa* or *levodopa/carbidopa* to increase the availability of these drugs in the brain [36,37].

**Figure 13:**
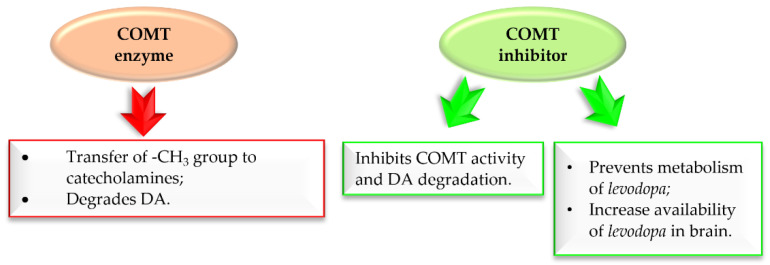
Action of COMT enzyme and COMT inhibitor in Parkinson’s disease.

Figure 13 shows the major COMT enzyme and COMT inhibitor activity associated with the pathogenesis of PD [33,34,35,36,37]. Thus, COMT inhibitors are a crucial approach in the treatment of PD.

**Figure 14:**
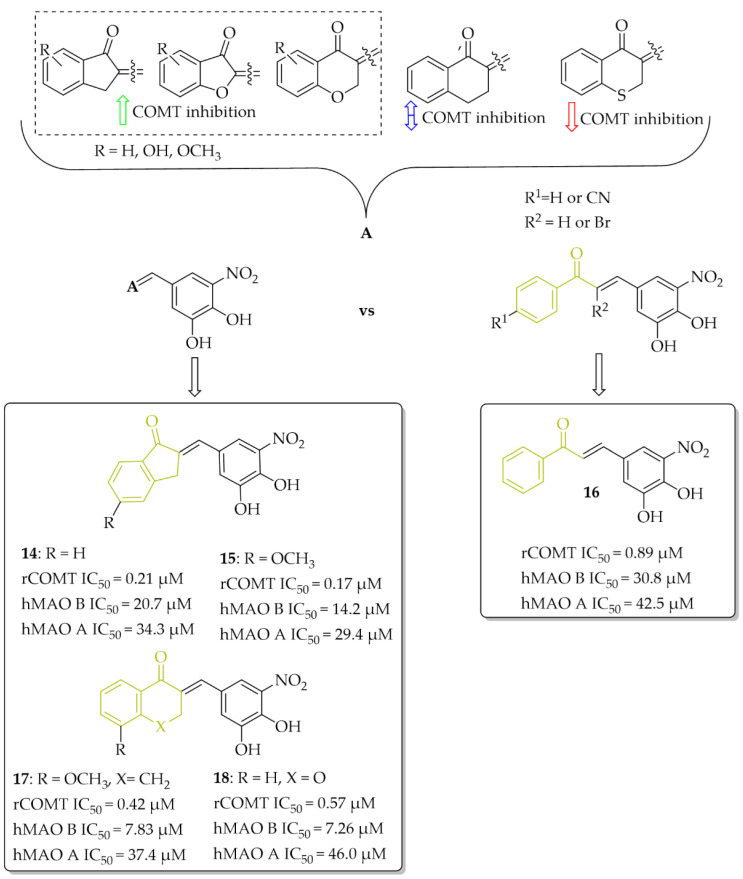
Dual COMT and MAO B inhibitors synthesised by de Beer et al. and the most potent compounds in this series [37].

Research to develop novel COMT inhibitors that also act on hMAO B using the chalcone structure was undertaken by de Beer et al. [37]. They synthesised a series of ring closure analogs of chalcones (14 compounds): 1-tetralone, 1-indanone and related derivatives, as well as chalcones (three compounds) with a nitrocatechol moiety as a **ring B** (Figure 14). Inhibition data showed that all compounds inhibited COMT enzyme activity with IC_50_ < 1 µM (except 4-thiochromanone derivative: IC_50_ = 1.69 µM). COMT enzymes were obtained from homogenates of rat liver tissue. The highest COMT inhibition showed 1-indanone derivatives, compound **14** and **15** (Figure 14), with an inhibitory activity (IC_50_) of 0.21 µM and 0.17 µM, respectively. In comparison with chalcone derivative **16** (Figure 14) with an IC_50_ of 0.89 µM, compounds had better COMT inhibitory activity. All tested compounds exhibited very weak hMAO B and hMAO A inhibitory activity (IC_50_ > 7 µM). The most promising dual COMT-MAO B inhibitors were compounds **17** and **18** (Figure 14) with an IC_50_ of 0.42 µM and 0.57 µM respectively for COMT, and IC_50_ of 7.83 µM and 7.26 µM respectively for hMAO B. Generally, the introduction of the nitrocatechol group provides a high capacity for COMT inhibition but results in very weak hMAO B inhibition [37].

**Figure 15:**
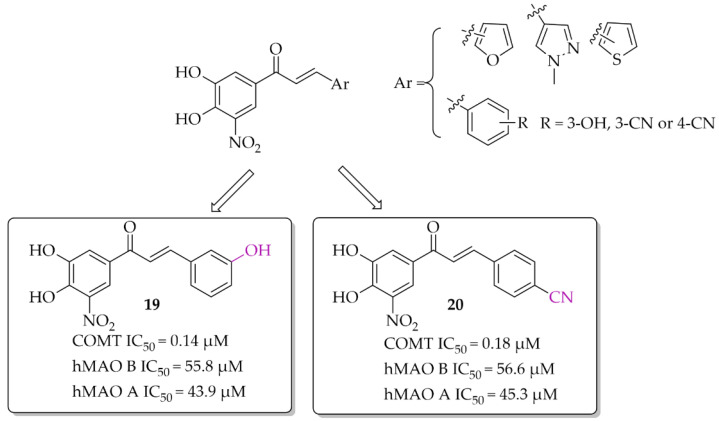
Dual COMT and MAO B inhibitors synthesised by Hitge et al. and the most potent compounds in this series [39].

A series of chalcones (nine compounds) with a nitrocatechol moiety as a **ring A** was obtained by Hitge et al. [39]. Inhibitory activity of compounds on COMT (using soluble fractions of rat liver tissue) and hMAO B was investigated. The recorded IC_50_ values for COMT inhibition were below 0.30 μM and for hMAO B IC_50_ values were from 42 μM to 72 μM. All compounds were also tested for hMAO A inhibition and the results showed comparable inhibitory activity of chalcones to hMAO B inhibition (hMAO A: 43 μM < IC_50_ < 70 μM). Compounds **19** and **20** (Figure 15) are the most potent dual inhibitors in this series (COMT: IC_50_ = 0.14 μM for **19** and 0.18 μM for **20**; hMAO B IC_50_ = 55.8 μM for **19** and 56.6 μM for **20**). SAR analysis showed that all changes made in the **ring B** did not influence COMT inhibitory activity. Regarding hMAO B inhibition, observations were similar to de Beer et al. [37]; the nitrocatechol group is not beneficial in inhibiting this enzyme.

#### 2.3.2. Monoamine Oxidase B and Cholinesterase Inhibitors

**Figure 16:**
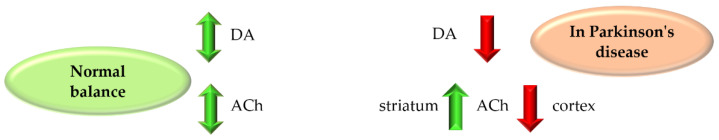
Normal and disturbed balance of dopamine (DA) and acetylcholine (ACh) in Parkinson’s disease.

Physiological interaction between DA and acetylcholine (ACh) allows keeping neuronal information. Loss of these interactions impairs motor and cognitive performance. Imbalance in dopaminergic and cholinergic transmission is observed in the striatum and cortex [40] (Figure 16).

In the striatum, a decrease in dopaminergic transmission is accompanied by an increase in cholinergic transmission. Hence, anticholinergic drugs (e.g., *benzatropine*, *biperiden*, *trihexyphenidyl*) improve motor function in patients. In contrast, a decrease in cholinergic transmission is observed in the cortex, which leads to cognitive dysfunction [40]. Cognitive impairments are observed in PD. As the disease progresses, this impairment increases. Currently, two types of cognitive impairments, based on severity and impact on daily living activities are observed in PD: mild (PD-MCI) and severe (PD dementia; PD-D) [41]. PD-MCI with subjective symptoms (attention deficit disorder, e.g., apathy) do not impair daily functioning [41], whereas PD-D with severe memory impairment, e.g., delusions, hallucinations, do not allow daily activities. It is believed that approximately 24–31% of patients have PD-D. Furthermore, observations show that the risk of disorder increases with time and more than 75% of patients with more than 10 years of disease progression develop dementia [41]. Currently, for dementia treatment in PD, cholinesterase inhibitor (ChEI) *rivastigmine* is used, but other drugs are also under investigation [41]. Gait disturbances and lack of balance are the main symptoms of PD. Initially, a shortening of the stride length and swinging of the shoulders are observed, which leads to a reduction in walking speed. As disease progresses, gait becomes more unstable, freezing, and falls may occur, which may be dangerous for the patient. This is caused, among other things, also by disturbances in cholinergic transmission. A decrease in cholinergic transmission leads not only to cognitive but also to gait disturbances. Moreover, scientific studies show that an increase in cognitive impairment leads to more frequent falls in PD. Improving cholinergic deficiency with ChEI may be a promising strategy in reducing gait disturbances. Clinical trials (phase 3—NCT04226248) are currently being conducted using *rivastigmine* as a ChEI to assess its effectiveness on reducing falls in people with idiopathic Parkinson’s disease [42]. Thus, ChEIs are a promising treatment of PD.

##### Phenothiazine-Based Chalcones

**Figure 17:**
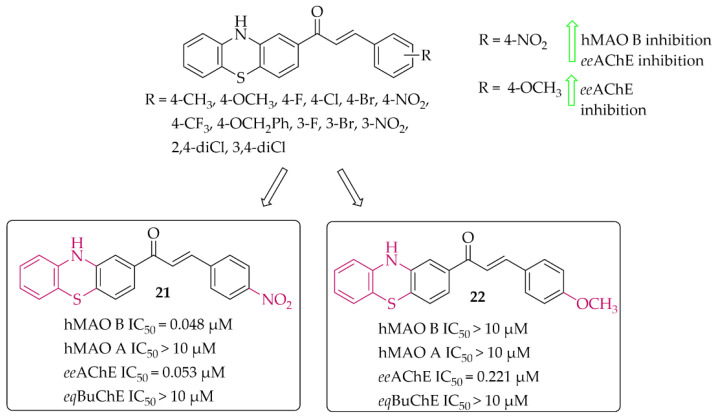
Phenothiazine-based chalcones and the most potent compounds in the series [43].

Yamali et al. [43] investigated 16 phenothiazine-based chalcones as potential inhibitors acting on hMAO B and AChE from electric eel (*ee*AChE). Phenothiazine moiety was as ring **A**. Ring **B** was substituted phenyl, tiophen, or benzo[1,3]dioxole. First, all compounds were screened in two concentrations (1 mM and 0.1 mM) for inhibitory potency for *ee*AChE, butyrylcholinesterase (from equine serum; *eq*BuChE), and hMAO B and hMAO A. Then, the most promising compounds were further evaluated to calculate IC_50_ values (two compounds for *ee*AChE and one for hMAO B). None of the compounds showed promising activity at 0.1 mmol against *eq*BuChE and hMAO A. The most active compound **21** (Figure 17) showed inhibitory activity for hMAO B with an IC_50_ value of 48 nM and *ee*AChE with an IC_50_ value of 53 nM. Structure–activity relationship analysis showed that compounds having a substituent in the *para* position at the phenyl ring (**ring B**) are the most active. Shifting these substituents to the *ortho* or *meta* position resulted in a decrease in activity. The most promising substituents were 4-NO_2_ (dual target activity) and 4-OCH_3_ (AChE inhibition; compound **22**; Figure 17) [43].

##### Piperazine-Based Chalcones

**Figure 18:**
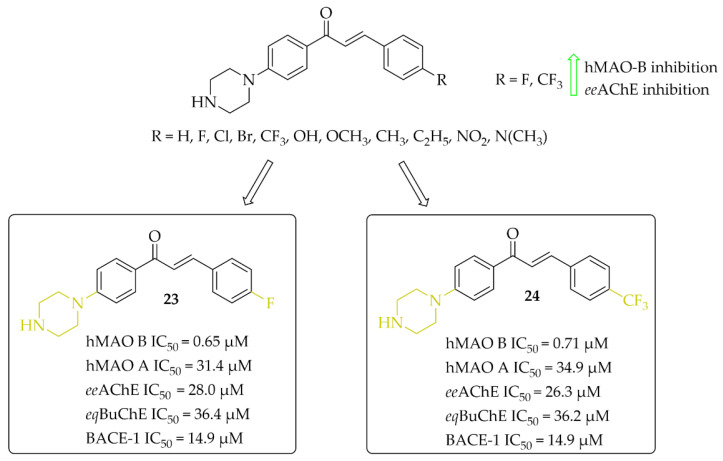
Piperazine-based chalcones as multitarget inhibitors and the most potent compounds in this series [44].

Eleven multitarget piperazine-based chalcones as inhibitors for the treatment of neurological disorders were described by Mathew et al. [44]. Ring **A** was phenylpiperazine and ring **B** was substituted phenyl (Figure 18). Compounds were tested for inhibitory activity towards MAOs, ChEs and BACE-1 (β-site amyloid precursor cleaving enzyme 1). First, compounds were screened at 10 μM concentration for all biological targets. Then, for the most promising chalcones, IC_50_ values were determined. All compounds showed good hMAO B inhibitory activity with IC_50_ values in a low (sub)micromolar range (0.60 μM < IC_50_ < 8 μM) and more than 10 times lower activity at hMAO A (IC_50_ > 27 μM). In contrast, all chalcones showed weak inhibition of cholinesterases (IC_50_ > 26 μM; except the compound with a 4-CH_3_ substituent: *ee*AChE IC_50_ = 8.77 μM). Whereas, some compounds moderately inhibited BACE-1 (IC_50_ > 6 μM). All results showed that two compounds **23** (with 4-F) and **24** (with 4-CF_3_) (Figure 18) are especially interesting, and thus they were subjected to further evaluation. Kinetics and reversibility studies of hMAO B inhibition confirmed that compounds **23** and **24** are competitive (K*_i_* = 0.63 μM and 0.53 μM respectively) and reversible inhibitors. Whereas, molecular docking studies showed that the phenyl ring with 4-F (**23**) and 4-CF_3_ (**24**) substituents faced the FAD of MAO B and formed π-π interactions with the corresponding amino acid residues [44].

##### Morpholine-Based Chalcones

**Figure 19:**
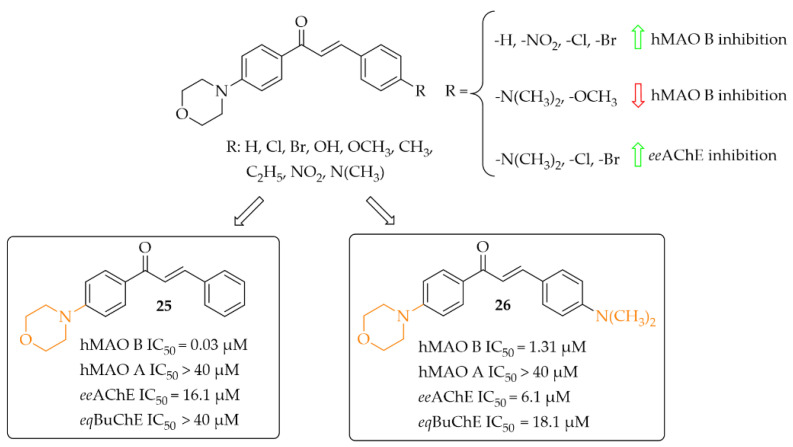
Morpholine-based chalcones and the most potent compounds in the series [45].

Nine morpholine-based chalcones were synthesissed by Sasidharan et al. [45]. Ring **A** was phenylmorpholine and ring **B** was substituted phenyl (Figure 19). Compounds were tested at 1 μM for hMAO B and at 10 μM for hMAO A, *ee*AChE, and *eq*BuChE. The study showed that most compounds exhibited strong inhibition of hMAO B (IC_50_ from 0.03 μM to 1.31 μM) and moderate to weak inhibition of *ee*AChE (IC_50_ > 6.1 μM). Moreover, compounds weakly inhibited hMAO A (IC_50_ > 7.1 μM) and *eq*BuChE (IC_50_ > 18.1 μM). Structure–activity relationship analysis showed that unsubstituted compound **25** (Figure 19) had the highest hMAO B inhibitory activity in the whole series (IC_50_ = 30 nM). An introduction of substituents such as -NO_2_, -Cl, and -Br improved the hMAO B inhibitory activity in comparison with other substituents (Figure 19). In contrast, the opposite effect was observed when -N(CH_3_)_2_ and -OCH_3_ groups were substituted. For *ee*AChE inhibition, an introduction of lipophilic groups such as -N(CH_3_)_2_, -Cl, and -Br enhanced inhibitory activity. The most active *ee*AChE inhibitor was compound **26** (Figure 19) with an IC_50_ of 6.1 μM. Further studies showed that compound **25** was a reversible mix-typed hMAO B inhibitor (K*_i_* = 18 nM), whereas compound **26** proved to be a reversible competitive *ee*AChE inihibitor (K*_i_* = 2.52 μM). In PAMPA assay both compounds showed high CNS permeability with Pe (×10^−6^ cm/s) values 16.34 and 14.44 respectively. ROS induced by 100 μg/mL of H_2_O_2_ in Hela cells was inhibited by 40 μg/mL of compound **25** and **26**. The strongest effect was observed for compound **25**. Thus, these two compounds are promising structures for further modification: compound **25** as a selective hMAO B inhibitor, and compound **26** as a dual-acting MAO B and AChE inhibitor [45].

##### Aldoxime Ethers

**Figure 20:**
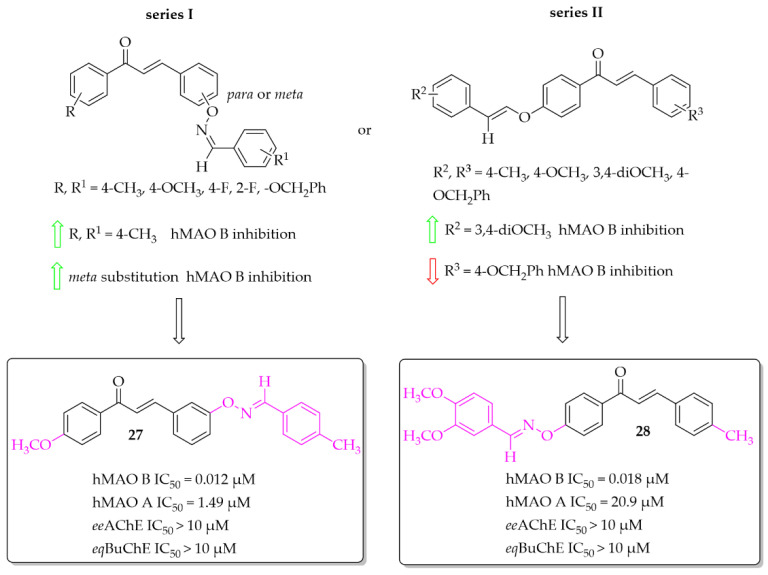
Aldoxime ethers and the most potent compounds in these series [46].

Compounds containing oxime ethers exhibit a variety of pharmacological activities, including antibiotic, antifungal, or anti-inflammatory properties [46]. Oh et al. [46], as a continuation of previous work [47], obtained 19 aldoxime chalcone ethers (ACE). The compounds were obtained by C-O coupling and their inhibitory activity towards hMAO B, hMAO A, ChEs and BACE-1 was evaluated. Two series of ACE were designed: series I (9 compounds) with a benzaldoxime group as the **B** ring, and series II (10 compounds) with a benzaldoxime group as the **A** ring. In the I series results showed that both the position of the benzaldoxime (*para* or *meta*), its substitution on the **B** ring, and the type of substituent on the **A** ring were crucial for increasing the inhibitory activity of the compounds towards hMAO B. From this series, compound **27** (Figure 20) had the strongest inhibitory activity of hMAO B with an IC_50_ of 12 nM [46]. Transferring the benzaldoxime group from the **B** ring to the **A** ring (series II) caused decreased hMAO B inhibition, in comparison with previously described compounds. The highest inhibitory activity among those compounds was compound **28** (Figure 20) with an IC_50_ of 18 nM [46]. None of compounds from these two series had nanomolar inhibitory activity for hMAO A. The most potent was compound **27** (Figure 20) with an IC_50_ of 1.49 μM. Moreover, none of the compounds tested showed any ability to inhibit ChEs and BACE-1 in a low micromolar range (IC_50_ > 10 μM) [46].

##### Hydroxychalcones

**Figure 21:**
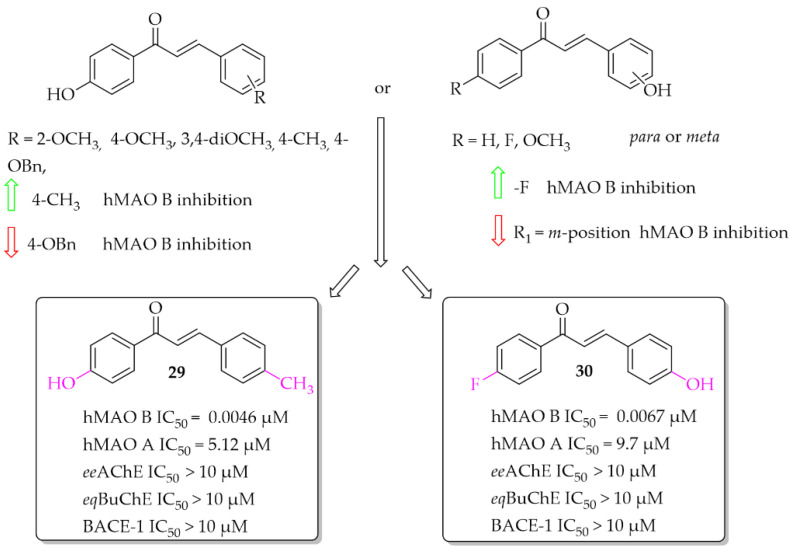
Hydroxychalcones and the most potent compounds in this series [46].

Eleven hydroxychalcones were synthesised by Oh et al. [46] and investigated for hMAOs, ChEs, and BACE-1 inhibition. A hydroxyphenol was either as ring **A** or the ring **B**. Activity was influenced not only by the position of the -OH group (ring **A** or **B**), but also by the type of substituent in the second ring. Too-small series of compounds did not allow explicit indication that the presence of -OH group in ring **A** was more favourable than in ring **B**, although results for two analogous compounds suggested it. None of the compounds showed effective inhibition of MAO A, ChEs, and BACE-1. The most potent as hMAO B inhibitors in the whole series were compounds **29** and **30** (Figure 21) with IC_50_ values of 4.6 nM and 6.7 nM respectively. Both compounds proved to be reversible competitive inhibitors of this enzyme (K*_i_* = 1.3 nM for compound **29**; K*_i_* = 3.6 nM for compound **30**). Moreover, all synthesised compounds exhibited higher inhibitory activity for hMAO B than for hMAO A (selectivity index >16), showing good selectivity. Molecular docking studies to hMAO A and hMAO B confirmed high activity and selectivity of those compounds [46].

##### Coumarin-Chalcones

**Figure 22:**
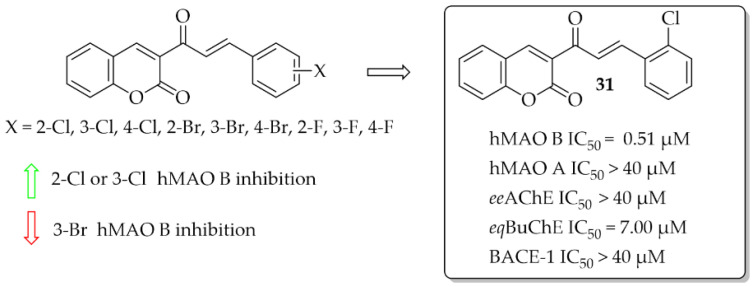
Coumarin-chalcones with the most potent compound in this series [48].

A series of 10 coumarin-chalcones was described by Rehuman et al. [48]. Unsubstituted coumarin was as ring **A**, whereas halogenated phenyl moiety was ring **B**. Compounds were tested for inhibitory activity towards hMAOs, ChEs, and BACE-1. First, all compounds were screened at 10 μM for all biological targets. The obtained results showed that all compounds had inhibitory activity for hMAO B in a (sub)micromolar range and only two in a micromolar range for *eq*BuChE. None of the compounds showed promising inhibition of hMAO A, *ee*AChE, and BACE-1 (IC_50_ > 40 μM). The most potent was compound **31** (Figure 22) inhibiting hMAO B with an IC_50_ of 0.51 μM and *eq*BuChE with an IC_50_ of 7.00 μM. Compound **31** proved to be a competitive and reversible inhibitor of both these enzymes (hMAO B: K*_i_* = 0.53 μM; *eq*BuChE: K*_i_* = 2.84 μM). Toxicity studies on Vera cells showed that compound **31** was not toxic to these cells at concentrations up to 100 μg/mL. Furthermore, compound **31** exhibited neuroprotective effects by attenuating the toxic effects induced by H_2_O_2_ treatment (100 μg/mL of 30% H_2_O_2_ for 10 min) [48].

## 3. Targeting *α*-Synuclein

**Figure 23:**
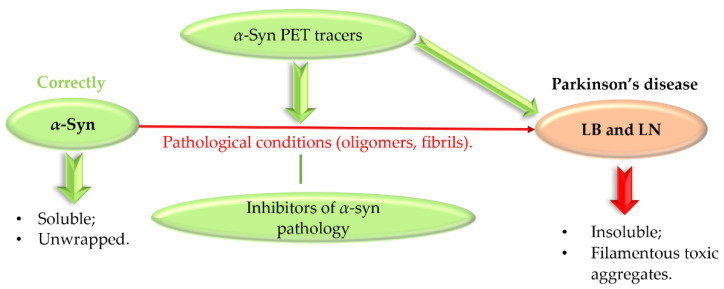
Formation of insoluble *α*-synuclein aggregates and a role of *α*-synuclein PET tracers and inhibitors. PET—positron emission tomography, LB—Lewy bodies, LN—Lewy neurites.

Abnormal protein aggregation is associated with many NDs. The main pathological feature of PD is the formation of Lewy bodies (LB) and Lewy neurites (LN), which contain the presynaptic protein *α*-synuclein (*α*-syn) [49,50]. Normally, *α*-syn exists in a soluble unfolded state, but under pathological conditions it undergoes abnormal folding and aggregation into toxic aggregates (Figure 23) [50].

Toxic forms of *α*-syn, especially oligomers, can cause oxidative stress, membrane penetration, and synaptic and mitochondrial dysfunction [49]. Although *α*-syn oligomers are toxic species and contribute to disease pathogenesis, *α*-syn fibrils play a key role in disease spread and progression [51]. Thus, *α*-syn, as a key pathogenic protein in PD, is an interesting therapeutic target. Recently, Grosso Jasutkar et al. [14] reviewed therapeutic approaches connected with *α*-syn, as well as compounds in the pipeline. The investigated strategies include:

- Removing of pathological aggregates from the brain—immunisation bullet;
- Reduction of *α*-syn expression;
- Inhibition of *α*-syn aggregation;
- Enhancing clearance and degradation of *α*-syn;
- Other genes/proteins involved in synucleinopathies;
- Decreasing synaptic activity.

So far, drugs for PD can only alleviate symptoms, so it is important to start treatment as soon as possible. Imaging of *α*-syn toxic aggregates (oligomeric, fibrillic) could be a useful tool not only for early diagnosis of disease but also for its development. Recently Xu et al. [52] and Korat et al. [53] reviewed the progression of this strategy. Development of radiotracers for positron emission tomography (PET) targeting *α*-synucleinopathies is very challenging. There are certain requirements that should be met [53]:

- Very high affinity for *α*-syn (even in the subnanomolar range);
- High selectivity of imaging *α*-syn (quantify *α*-syn in the presence of higher density of Aβ or/and tau proteins);
- Good permeability to cross not only BBB but cell membranes;
- Low binding to off-targets (>500 nM);
- Good metabolic stability.

In the last 5 years, chalcones were described as promising tools for imaging *α*-syn fibrillation and aggregation [53,54].

### 3.1. Imaging Probes of α-Syn Inclusions

**Figure 24:**
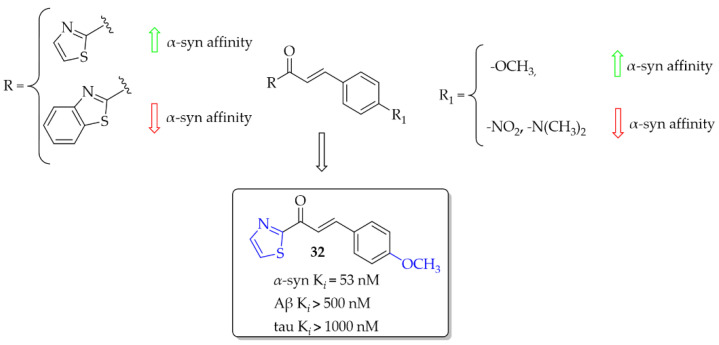
Chalcones with affinities for α-syn fibrils and the most active compound in the series [54].

(Benzo)thiazole chalcones and their heterocyclic isosteres were described by Hsieh et al. [54] as compounds with affinity for *α*-syn fibrils. Since beta-amyloid (Aβ) plaques and tau proteins are frequently observed in the brains of PD patients, the affinity for these proteins was also checked. All nine compounds were evaluated in vitro in thioflavin T (ThT) competition binding assays. When ring **A** was a thiazole moiety, the affinity for *α*-syn fibrils was better than for benzothiazole analogues. Moreover, compounds also showed higher selectivity vs. Aβ and tau proteins. An increase in the affinity for *α*-syn fibrils was also observed when the -OCH_3_ substituent was substituted at the **B** ring of the chalcone. The most potent compound **32** (Figure 24) had an affinity for *α*-syn fibrils with K*_i_* of 53 nM and good selectivity for other proteins (K*_i_* > 500 nM). Conversely, when -NO_2_ or -N(CH_3_)_2_ groups were present, the decrease of the affinity for *α*-syn was observed as well as decrease in the selectivity. Molecular docking studies showed that the intramolecular distance between hydrogen bond acceptors may be one of the factors influencing the binding affinity with the *α*-syn protein [54]. However, compounds were not radiolabelled and evaluated in vivo to check their utility.

**Figure 25:**
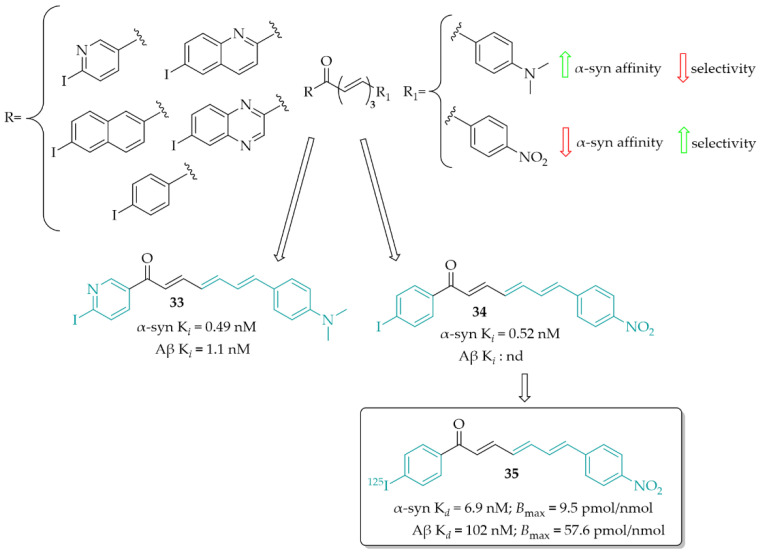
Chalcones as *α*-syn imaging probes and the most active componuds in this series; nd—not determined [55].

Designing *α*-syn detection probes was also described by Kaide et al. [55]. This work was a continuation of previous work in this field [56]. Now, a series of chalcones with different aryl groups in the ring **A** and 4-(dimethylamino)phenyl or 4-nitrophenyl (as the ring **B**) were designed and synthesised. Next, binding affinity of compounds towards *α*-syn was evaluated in the ThT competitive binding assay. Then, in the same assay, binding affinity for Aβ was evaluated. In vitro studies showed that the aryl groups positively affected the affinity for inhibiting fibre aggregation of recombinant *α*-syn (K*_i_* ranging from 0.49 to 13 nM). Among the series with the 4-(dimethylamino)phenyl group, compound **33** (Figure 25) showed the highest affinity for *α*-syn (K*_i_* = 0.49 nM) but also the highest affinity for Aβ (K*_i_* = 1.1 nM). In the 4-nitrophenyl series, the most potent was compound **34** (Figure 25) with a K*_i_* of 0.52 nM. In this series, none of compounds showed affinity for Aβ aggregates. These results were also confirmed in human aggregates. 4-(Dimethylamino)phenyl derivatives visualised both *α*-syn and Aβ aggregates whereas, in the 4-nitrophenyl series compounds recognised only human *α*-syn aggregates. Thus, compound **34**, as the most promising, was chosen for ^125^I labelling reaction and further studies (compound **35**; Figure 25). In a saturation binding assay, compound **35** bound to *α*-syn aggregates with a high affinity (K*_d_* = 6.9 nM) and maximum of binding sites (B_max_) of 9.5 pmol/nmol protein, whereas for Aβ aggregates K*_d_* it was 102 nM and B_max_ was 57.6 nM, respectively. Pharmacokinetic studies in vivo showed high stability in murine plasma and moderate uptake in the mouse brain. Thus, compound **35** is a promising lead structure in the search for radio-labelled tracers for imaging *α-*syn inclusions [55].

### 3.2. α-Synuclein Inhibitor—Natural Chalcone Derivative—Butein

**Figure 26:**
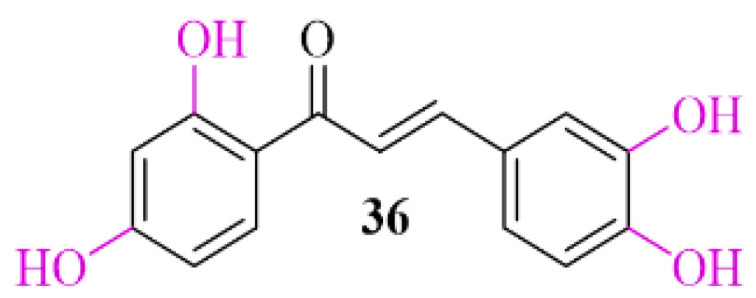
Chemical structure of butein (**36**).

Natural chalcones show the ability to inhibit *α*-syn aggregation [57]. Butein (**36**; Figure 26), a natural chalone, is present in many plants. Extract of these plants are commonly used to treat different diseases (e.g., infections, inflammations, cancer) in Asian countries [58]. Recently, Tinku et al. [59] investigated the ability of butein to inhibit *α*-syn aggregation. Moreover, researchers tried to explain the mechanism of this inhibition by spectroscopic, microscopic, and computational methods. Undertaken studies showed reduction in formation of *α*-syn aggregates in the presence of butein. Molecular dynamic and docking studies indicated that butein binds to *α*-syn selectively and forms a multipoint contact via hydrogen bonding and hydrophobic interactions. This contact reduces the flexibility of *α*-syn, which delays misfolding and structural transition [57].

## 4. Adenosine A_2A_ and A_1_ Receptor Antagonists

**Figure 27:**
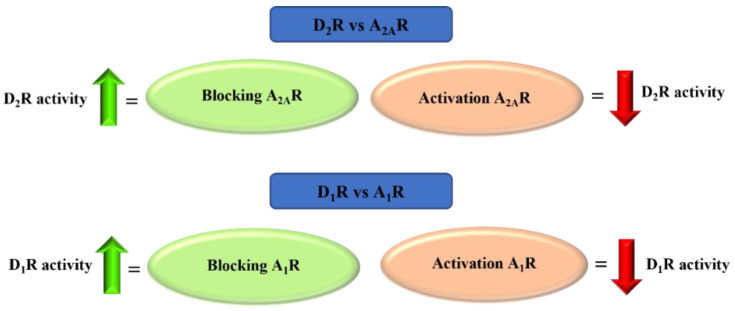
Adenosine A_2A_ and A_1_ receptors—antagonism of action with dopamine D_2_ and D_1_ receptors.

Adenosine is an endogenous nucleoside which takes part in many physiological and pathophysiological processes. It exerts its biological effect by activating four adenosine receptors: A_1_, A_2A_, A_2B_, and A_3_ [60]. Adenosine plays the opposite role to DA in the brain. At the level of GABAergic neurons, there is an antagonistic interaction between adenosine receptors (AR) and dopamine receptors. AR activation causes acute DA reduction or DA receptor blockade. AR agonists inhibit, whereas AR antagonists potentiate, DA receptor-activating effects. This antagonism results from the existence of selective interactions between specific adenosine and DA receptor subtypes in the striatum, among others, and adenosine A_2A_ receptor (A_2A_R) and dopamine D_2_ receptors (D_2_R) in the striopallidal neuron and between adenosine A_1_ receptor (A_1_R) and dopamine D_1_ receptors (D_1_R) in the striatonigral-strioentopeduncular neuron (Figure 27) [61,62,63,64].

AR ligands have attracted increasing interest in drug design and development for the treatment of neurodegenerative diseases. Recently Matthee et al. [65] reviewed heterocyclic structures related to chalcones as AR ligands with potential utility for the treatment of CNS diseases. In this review, we direct particular attention toward chalcones as A_1_R and A_2A_R receptor antagonists that may aid in the therapy of PD treatment.

### 4.1. Aniline-Based Chalcones

**Figure 28:**
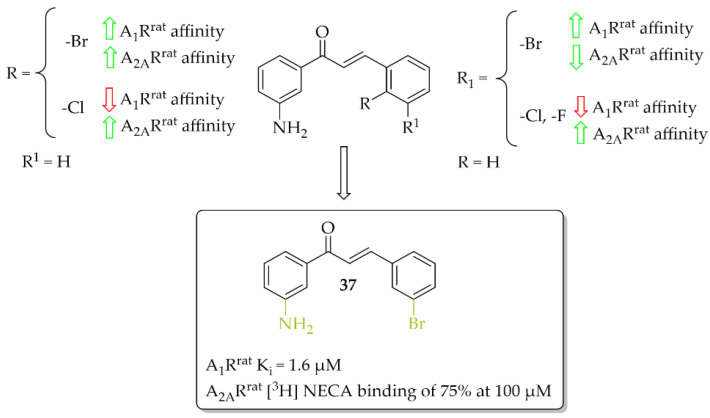
Aniline-based chalcones and the most active compound in the series [66].

A series of 27 amino-substituted chalcones was synthesised by van Rensburg et al. [66] and the effect of the substituent (e.g., halogen) and the position of this substituent on the **B** ring of the chalcone on the affinity for the rat A_1_R (rat whole membranes) and rat A_2A_R (rat striatal membranes) was investigated. The degree and type of binding affinity to these receptors was determined in a radioligand binding and a guanosine triphosphate (GTP) shift assays. As radioligands, 1,3-[^3^H]-dipropyl-8-cyclopentylxanthine ([^3^H]DPCPX) for A_1_R^rat^ and 5′-*N*-ethylcarboxamido [^3^H]adenosine ([^3^H]NECA) for A_2A_R^rat^ were used. Only for four compounds were IC_50_ values for rat A_1_R evaluated and these compounds showed micromolar affinity below 10 µM. Compound **37** with the 3-amino substituent at the ring **A** (Figure 28) proved to be the strongest selective antagonist of A_1_R^rat^ with a K*_i_* of 1.6 µM as determined by a GTP shift assay. This compound had a low relative affinity for A_2A_R^rat^ (specific binding [^3^H] NECA of 75% at 100 µM). Structure–activity relationship analysis showed that the amino group in the *meta* position of the **A** ring of chalcone and the bromine atom in the *meta* position of the **B** ring of chalcone played key roles in the affinity for A_1_R [66].

### 4.2. Chalcone Hybrids

#### 4.2.1. Benzofuran Derivatives

**Figure 29:**
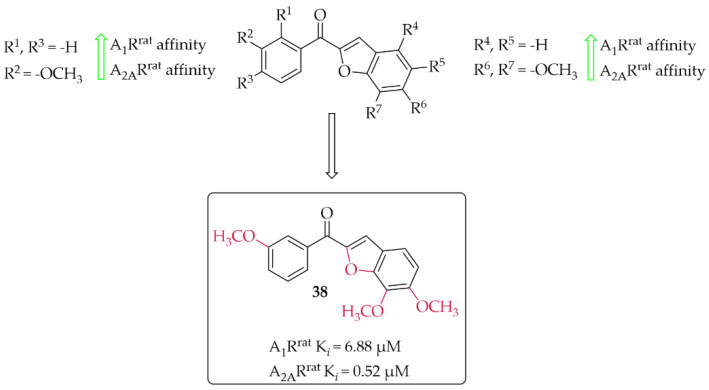
2-Benzoyl-1-benzofurans as adenosine A_1_ and A_2A_ receptor ligands and the most potent compound in this series [67].

A series of 14 methoxy substituted 2-benzoyl-1-benzofurans was synthesised and evaluated for A_1_R and A_2A_R affinity by van Rensburg et al. [67]. The affinity was determined in a radioligand binding assay on the rat whole brain membranes (A_1_R^rat^) and rat striatal membranes (A_2A_R^rat^). Structure–activity relationship analysis showed that the position of the substituent (methoxy group) on both the **A** and **B** ring of the chalcone derivatives has the effect of increasing or decreasing the affinity for both A_1_R^rat^ and A_2A_R^rat^. Only five compounds showed promising affinity for A_1_R^rat^ in low micromolar values (5 µM < K*_i_* < 9 µM) and one compound for A_2A_R^rat^ (K_i_ < 1 µM). The *meta* position on the **A** ring and the *para* position on the **B** ring of the chalcones appeared to be most beneficial, on increasing the affinities for both A_1_R^rat^ and A_2A_R^rat^. Compound **38** (Figure 29) proved to be the only compound that showed dual affinity to A_1_R^rat^ (K*_i_* = 6.88 µM) and A_2A_R^rat^ (K*_i_* = 0.52 µM). In addition, this compound had low cytotoxicity (IC_50_ > 100 µM in Vero cells) and good drug-like properties [67]. Moreover, the activity profile of **38** was determined only for interactions with the A_1_R^rat^. The GTP shift assay showed that this compound acted as an antagonist. Based on structural similarity, other 2-benzoyl-1-benzofuran derivatives are expected to behave as A_1_R antagonists, too [67].

#### 4.2.2. Indanone Derivatives

**Figure 30:**
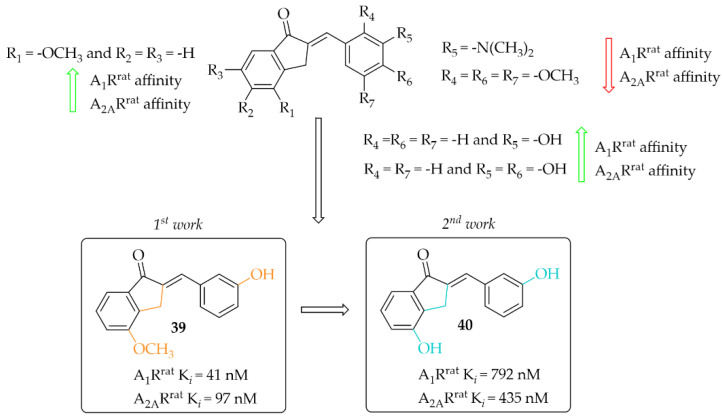
2-Benzylidene-1-indanone as adenosine A_1_ and A_2A_ receptor ligands and the most potent compounds in this series [68,69].

Van Rensburg et al. in two other works described 2-benzylidene-1-indanone derivatives as A_1_R and/or A_2A_R ligands [68,69]. The effect of substituents (-OH, -OCH_3_, or -N(CH_3_)_2_) and their position on the **A** and/or **B** ring of the chalcone moiety was investigated. In the first work, 12 compounds [68] were described whereas in the second work 14 compounds were described [69]. First, authors changed the position of substituents in both **A** (only methoxy group) and **B** rings whereas in the second study they further investigated these changes with substituents in the best position from the first studies. Affinities for rat receptors A_1_R and A_2A_R were evaluated in the radioligand binding assays as described in previous work [67]. In the first study [68], SAR analysis showed a positive effect of the -OCH_3_ substitution on the **A** ring on the affinity. In addition, substitution with the -OH group on ring **B** resulted in increased affinity for both tested adenosine receptors. The most potent compound this series (compound **39**; Figure 30) had nanomolar affinities for A_1_R^rat^ (K*_i_* = 41 nM) and A_2A_R (K*_i_*^rat^ = 97 nM). The activity profile of **39** was determined only for interactions with the A_1_R^rat^. The GTP shift assay showed that this compound acted as an antagonist, as no significant rightward shift of the binding curve was observed in the presence of GTP [68].

In the other work, van Rensburg et al. [69] described a series of 2-benzylidene-1-indanones (14 compounds) and 2-benzylidene-1-tetralones (five compounds) differing in the type of substituent (-OH or -OCH_3_ group) and its position on the **A** and/or **B** ring of the chalcone moiety. SAR analysis showed that the presence of a hydroxyl group (on the **A** and **B** ring) promotes increased affinity for both adenosine receptors. In the case of a methoxy group, it causes a decrease in affinity for A_1_R and A_2A_R when located on the **B** ring. The most active compound of this series turned out to be compound **40** (Figure 30), which showed affinities for both adenosine receptors in submicromolar ranges (A_1_R^rat^ K*_i_* = 0.79 µM; A_2A_R^rat^ K*_i_* = 0.44 µM) [69]. Intrinsic activity profile studies were performed only for compound **40** and interactions with the A_1_R. The GTP shift test confirmed the activity of **40** as an A_1_R antagonist.

#### 4.2.3. Tetralone Derivatives

**Figure 31:**
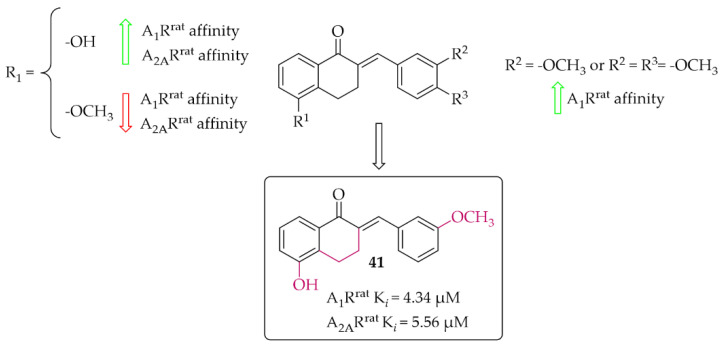
Tetralone-based chalcones and the most potent compound in the series [69].

The synthesis and affinity for A_1_R and A_2A_R receptors of chalcones based on a 2-benzylidene-1-tetralone moiety was also reported by van Rensburg et al. [69]. Again, an effect of a position of a hydroxyl and a methoxy substituent on the **A** and/or **B** ring of chalcone on the A_1_R and A_2A_R affinity was studied. Biological activity for rat A_1_R and A_2A_R was evaluated in the radioligand binding assays. The structure–activity relationship analysis showed that the hydroxyl group on the **A** ring has a positive effect on increasing the affinity, while the methoxy group has the opposite effect. In the case of substituents on the **B** ring, an increase in the affinity for A_1_R of the compound that had a methoxy group in the *meta* position was observed. The most active compound of this series was compound **41** (Figure 31) with values A_1_R^rat^ K*_i_* = 4.34 µM and A_2A_R^rat^ K*_i_* = 5.56 µM. The type of binding affinity of **41** for only A_1_R was determined by a GTP shift assay. The results suggested that this compound was an antagonist as the binding curves in the presence of GTP were almost unaffected and the calculated GTP shifts were approximately 1.

#### 4.2.4. Coumarin Derivatives

**Figure 32:**
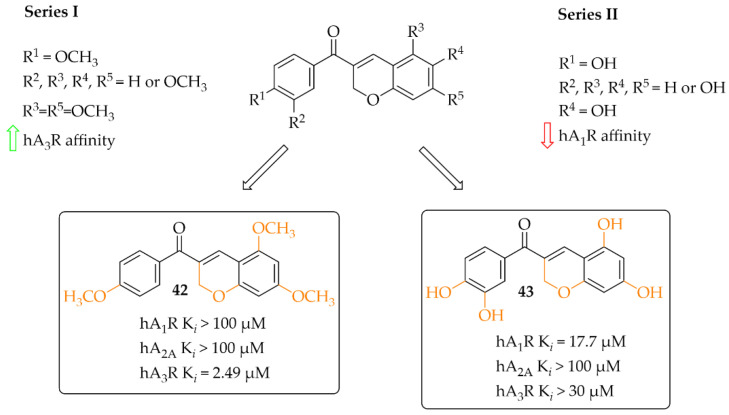
Coumarin-based chalcones with the most potent compounds in the series [70].

A small series (eight compounds) of coumarin-chalcone hybrids with a methoxy (series 1) or a hydroxyl (series 2) substituent was synthesised by Vazquez-Rodriguez et al. [70]. Compounds were tested in radioligand binding assays (human A_1_R, Human A_2A_R and human A_3_R) and adenylyl cyclase assay (A_2B_R) for affinity to adenosine receptors. None of compounds showed affinity in a low micromolar range for A_2A_R (K*_i_* > 100 µM) and A_2B_R (K*_i_* > 10 µM). Four compounds had affinity for hA_1_R (17 µM < K*_i_* < 55 µM) and three for hA_3_R (2 < K*_i_* < 35 µM). Generally, active compounds were more potent A_3_R than A_1_R ligands. The most potent compounds (**42** and **43**) in both series are shown in Figure 32. Unfortunately, information on the type of intrinsic activity with the receptors tested was not included. Further, molecular modelling studies were conducted to explain the observed differences in binding affinity and selectivity (A_1_R/A_3_R), resulting from different hydrophobic (A_3_R) and hydrophilic (A_1_R) interactions of the respective receptor region with ligands [70].

## 5. Targeting Oxidative Stress and Neuroinflammation

Neuroinflammation plays a crucial role in a pathogenesis of ND including PD [71,72]. Dopamine released from synaptic vesicles into the synaptic cleft or cytosol undergoes various reactions (enzymatic and non-enzymatic) during which free radicals (H_2_O_2_, ∙O_2_^−^, and ∙OH^−^) are produced. Oxidative stress contributes to neuronal loss in many disease states and also during normal ageing. Nitrative stress associated with nitric oxide (∙NO) which is produced by nNOS and iNOS during neuroinflammation also contributes to dysfunction of dopaminergic neurons. Redox imbalances lead to activation of pathways that are normally inactive, causing cell damage and/or death [73]. These changes have a particular effect on *α*-synuclein, increasing its tendency toward aggregation [74]. Antioxidant activity leading to reduced oxidative stress may increase survival of dopaminergic neurons. Thus, compounds that affect signalling associated with neuroinflammation are an attractive target for the treatment of ND, among others.

### 5.1. The Nuclear Factor Erythroid 2–Related Factor 2 Activation

**Figure 33:**
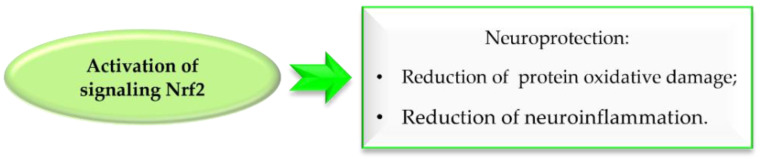
Effect of Nrf2 signalling activation in Parkinson’s disease.

Reactive oxygen species (ROS) formed during DA metabolism decrease intracellular oxidant (glutathione) levels and increase iron and calcium levels. Under normal conditions, the nuclear factor erythroid 2–related factor 2 (Nrf2), which is a transcription factor, is bound to the cytosolic inhibitory protein Keap-1. However, under oxidative stress or in the presence of electrolytes, Nrf2 is released and binds to antioxidant response elements (ARE). The gene expression of ARE containing antioxidant enzymes is induced by Nrf2. Thus, Nrf2 activation exhibits neuroprotective effects by reducing oxidative damage and nerve inflammation, which may aid in the fight against PD (Figure 33) [75,76]. Chalcones due to electrophilic double bond could covalently bind with cysteines in Keap1, leading to dissociation of the Keap1-related Nrf2, which is stored in the cell nucleus and activates enzymes and proteins involved in xenobiotic detoxicfication [76].

#### 5.1.1. Compound **KMS99220**

**Figure 34:**
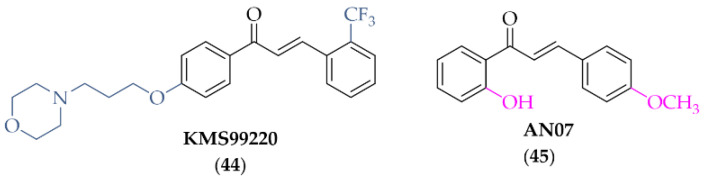
Chemical structure of **KMS99220** (**44**) and **AN07** (**45**) [77,78,79].

Lee et al. [77] described the synthesis and biological activity of a chalcone with a morpholine moiety (compound **44**; **KMS99220**) (Figure 34). This compound had high binding affinity to Nrf2 protein and increased the expression of antioxidant enzyme genes (NQO1, HO-1, and GCL). Moreover, it increased the expression of proteasome subunits (PSMB5, PSMB7, PSMB8, and PSMA1) and their protease activity and decreased *α*-syn aggregation. It had a good pharmacokinetic profile, metabolic stability, bioavailability, and non-toxicity in vivo up to doses of 2000 mg/kg. In a mouse model of PD with 1-methyl-4-phenyl-1,2,3,6-tetrahydropyridine (MPTP) as a toxin, oral administration of **KMS99220** (10 and 30 mg/kg) attenuated motor dysfunction, protected dopaminergic neurons, and induced Nrf2 signalling [77]. Further studies with **KMS99220** by Lee et al. [78] showed activation of AMP protein kinase (AMPK) and heme oxygenase-1 (HO-1) signalling by this compound, which induced anti-inflammatory effects. Next, this compound was tested in vivo at two doses (10 and 30 mg/kg) in the mice model of PD (with MPTP) to evaluate its anti-inflammatory effect [79] and the signalling pathways involved in this activity. The results showed that **KMS99220** inactivated kinases IKK, p38 MAPK, and JNK in the inflammatory signalling pathways. Thus, **KMS99220** is a promising candidate with neuroprotective properties for the treatment of neuroinflammatory diseases (including PD).

#### 5.1.2. Compound **AN07**

Chen et al. described biological activity of 2-hydroxy-4′-methoxychalcone (**AN07**; compound **45**; Figure 34) [80]. **AN07** exhibited antioxidant activity against lipopolysaccharide (LPS)-induced inflammation and RAW 264.7 macrophages. Analysis of the study showed that **AN07** inhibited the regression of ROS and reduced NO production, the regulatory enzyme iNOS, and cyclooxygenase-2 (COX-2). Furthermore, confirmation of its potential in the treatment of inflammatory disorders is that the compound enhances antioxidant regulation of Nrf2/HO-1 pathways. The results presented here also showed that **AN07** reduced methylglyoxal (MG)-induced apoptotic death and neurite damage in cells. This was associated with upregulation of IGF-1R, GLP-1R, and BDNF, activation of antioxidant Parkinsonism proteins (*parkin*, *pink1*, and *DJ1*), and inhibition of the Rho-associated protein kinase 2 (ROCK2)/phosphorylated LIM kinase 1 (p-LIMK1) pathway. These results hint at novel neuroprotective mechanisms of **AN07** against neuronal disorders [80].

#### 5.1.3. Chalcones Containing a Vinylsulfone Scaffold

**Figure 35:**
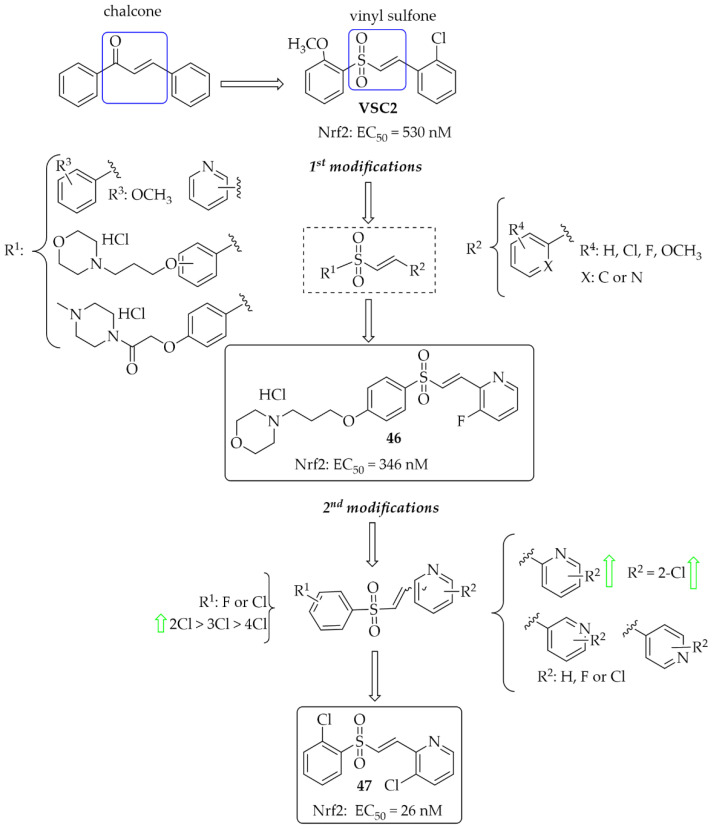
Design and structures of the most potent vinylsufonyl Nrf2 activators synthesised by Chen et al. [82,83].

A vinyl sulfone scaffold is structurally similar to the chalcone moiety. In 2014, Woo et al. [81] described the first such compounds as promising NrF2 activators protecting dopaminergic neurons both in vitro and in vivo studies. The compound **VSC2** (Figure 35) had the strongest effect and activated Nrf2 with an EC_50_ of 530 nM. As a follow-up to this work, Choi et al. published two papers that allowed obtaining highly potent NrF2 activators [82,83]. In the first work, to improve drug-like properties of **VSC2** a pyridine, a morpholine and a piperazine moiety were introduced [82] to a molecule. Thus obtained a series of 52 compounds was evaluated for activation-dependent Nrf2 translocation into the nucleus in a cell-based functional assay (Keap1-Nrf2 functional assay) as well as for ADME/Tox evaluation (CYP inhibition, cytotoxicity, microsomal stability). For the selected 15 compounds’ ability to induce expression of the HO-1 gene, ELISA (enzyme-linked immunosorbent assay) was also evaluated. Results showed that 14 compounds were potent Nrf2 activators with EC_50_ ≤ 350 nM. Most of them were also not cytotoxic at 10 µM. Inhibition of CYP enzymes (2C19, 2D6, 2D9 1A2, and 3A4) was performed using P450-Glo assay. Results showed that most compounds with the morpholine or piperazine ring (ring **A**) showed reduced ability to block the tested CYP450 enzymes. The same these group of compounds had good microsomal stability. Based on all in vitro results, compound **46** (Figure 35) was selected for further studies. In in vivo studies in the MPTP-induced mouse model of PD, compound **46** (at the dose of 20 mg/kg, p.o.) reduced motor dysfunction evaluated in vertical grid and coat-hanger behavioural tests. Moreover, it protected dopaminergic neurons and inhibited microglia activation. Thus, compound **46** showed potential ability for PD treatment. In the second work, Choi et al. [83] described a series of 61 compounds. The **A** ring was a substituted phenyl (with chlorine or fluorine), while the **B** ring was a differentially attached (un)substituted pyridine. Studies on the effect of halogenated vinylsulfones on Nrf2 activation showed that 31 compounds activated Nrf2 with EC_50_ below 1000 nM (including five compounds even below 100 nM). SAR analysis showed that in the **A** ring, the introduction of chlorine into the 2nd position was beneficial, while the mode of attachment of the pyridine ring (*ortho*, *meta*, or *para*) affected the activity. Derivatives with *ortho*-pyridine were the most active. The (Z) isomers had lower activity than the corresponding (E) isomers. The most promising compound, **47** (Figure 35), was subjected to further pharmacological studies, which confirmed its low cytotoxicity, high stability in human microsomes and plasma, a very high BBB permeability in the PAMPA assay (P_e_ = 65.16 × 10^−6^ cm/s), and low hERG toxicity (IC_50_ = 110 µM). Moreover, compound **47** induced the expression of the antioxidant genes HO-1, GCLC, GCLM, and SOD-1 at both mRNA and protein levels and inhibited the secretion of pro-inflammatory cytokines and enzymes in BV2 cells. At the dose of 30 mg/kg p.o., compound **47**, in the MPTP-PD mouse model, alleviated abnormal movements and improved behavioural function.

**Figure 36:**
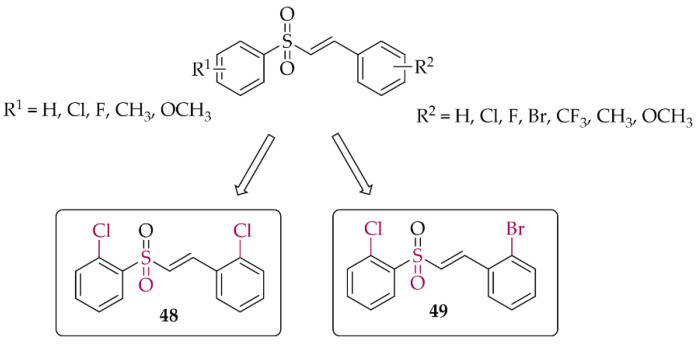
Structures of the most potent vinylsufonyl Nrf2 activators synthesised by Song et al. [84].

In 2021, Song et al. [84] described 47 chalcones with a vinylsulfone. First, cytotoxicity toward PC12 cells was evaluated in the MTT assay. A weak effect of the compounds at 50 µM was observed. Then, the neuroprotective effect of compounds on H_2_O_2_-induced lesions to PC12 cells was investigated. Of the whole series, two compounds (compound **48** and **49**; Figure 36) showed the strongest neuroprotection. Further studies were conducted to confirm the mechanism of their protective effect, i.e., effects on cytoprotective gene activation, Nrf2 translocation, or NrF2-ARE pathway activation. Promising results for these compounds led to their transfer to in vivo studies.

### 5.2. Inducible Nitric Oxide Synthase Inhibitors

**Figure 37:**
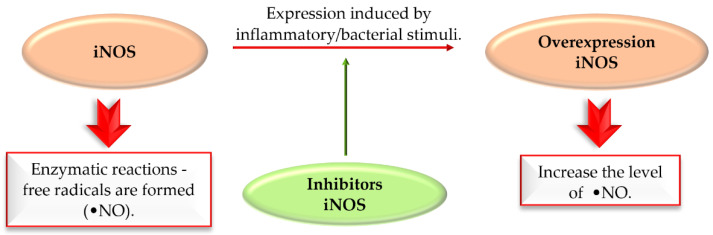
The action of iNOS enzyme and iNOS inhibitors in Parkinson’s disease.

Nitric oxide synthases (NOS) are enzymes which synthesise nitric oxide (NO). There are three isoforms of NOS: neuronal NOS (nNOS), inducible NOS (iNOS), and endothelial NOS (eNOS), reflecting the site of expression. Free radicals can be formed in enzymatic reactions catalysed by nitric oxide synthase (NOS). iNOS induced by inflammatory stimuli contribute to the production of NO. Overexpression of iNOS increases NO levels. Overproduction of NO may lead to cell death in various ND. Thus, selective inhibition of iNOS is an interesting approach to treat complex diseases (Figure 37) [85].

**Figure 38:**
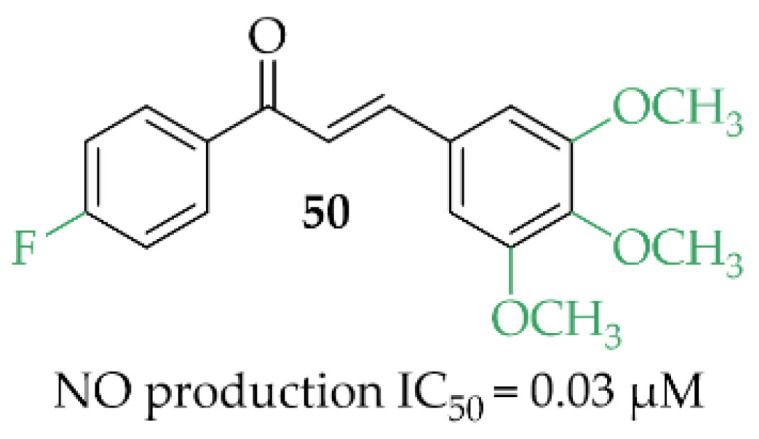
Structure of the most promising NO inhibitor [85,86].

A review article, written by Minhas et al. [85]*,* described many approaches to develop new iNOS inhibitors. One of the groups was chalcones and one of the most interesting structures was compound **50** (Figure 38), described by Rojas et al. [86] with NO inhibition of IC_50_ = 0.03 µM.

In vivo studies of compound **50** showed promising activity in an adjuvant-induced arthritis in rats at the dose of 25 mg/kg. This compound inhibited paw oedema and the levels of NO and prostaglandins PGE2 [87].

Unfortunately, in the last 5 years new iNOS inhibitors were not described in the literature.

## 6. Summary and Conclusions

Chalcones, as precursors to all known flavonoids, possess a broad spectrum of biological activity. They have been shown to have high inhibitory potential on the MAO B, COMT, AChE, iNOS enzymes, affinity for adenosine A_2A_ and A_1_ receptors, and high ability to inhibit *α*-syn aggregation and activate Nrf2 signalling. Such results can be obtained by simple modifications introduced into the **A** and/or **B** rings.

Recently described chalcones (during the last 5 years) with potential utility in PD are mostly hMAO B inhibitors. They can be mono- or multitarget ligands. The most common substituents in hMAO B inhibitors were halogens (especially chlorine and fluorine), hydroxyl, methoxyl, trifluromethyl, or nitro groups. Interestingly, the unsubstituted chalcone moiety (compound **1**; Figure 7) showed good hMAO B inhibitory activity (IC_50_ = 56 nM). In most cases, the **A** ring was changed to another heterocyclic ring. SAR analysis (not shown, from publications [27,28,29,30] and [43,44,45,48]) of these most common substituents in the **B** ring showed that the type of this **A** ring had a great influence on hMAO B inhibitory activity. The most potent hMAO B inhibitors were compounds with 1,3-benzodioxole or 1,4-benzodioxane as the **A** ring. Interestingly, the introduction of 1,4-benzodioxane as a **B** ring in the chalcone moiety resulted in decreased activity (compare compound **11**—Figure 10 vs. compound **8**—Figure 9) towards hMAO B but increased selectivity (decreased activity towards hMAO A). The compounds with an -OH substituent in the ring also had high inhibitory activity towards hMAO B. All these substituents were most favourable at the position *para*, both in the **A** and **B** rings. Changing the position of the substituent to other positions (*ortho* or *meta*) caused a decrease in this activity.

All compounds designed as MAO B inhibitors were also evaluated for selectivity towards MAO A. Non-selective inhibitors, particularly MAO A inhibitors, are characterised by an undesirable property—they can bind to MAO in a non- reversible manner, which translates into interactions with certain medications and foods. Eating meals that contain hypertensive amines may result in a sudden increase in blood pressure and the so-called cheese effect, which is dangerous for the body. Therefore, the selectivity of the described compounds was emphasised in the presented work. As the structures were obtained with the MAO B inhibitory activity in mind, it was not analysed exactly which substituents in the **A** and/or **B** ring and in which position caused an increase in MAO A activity. In most cases, the observed selectivity of the compounds was to be very high (>1000). Only in the case of oxidised chalcones [29], where 1,3-benzodioxole or 1,4-benzodioxoane was introduced as the **A** ring (compounds **7**–**9**), was comparable/high activity towards MAO A observed. When such a ring was used as the **B** ring, this effect was not observed (compare **11**—Figure 10 with **8**—Figure 9).

Analysing examples of chalcones as COMT inhibitors, it was noted that the nitro-catechol group favours COMT inhibitory activity, both as the **A** and **B** ring of the chalcone. In contrast, its presence decreased the hMAO B inhibitory activity (IC_50_ > 7 μM). All such compounds showed also hMAO A inhibition comparable to hMAO B (low selectivity < 10).

Structure–activity relationship analysis of chalcones, as probes to image the pathology of *α*-syn proteins and as inhibitors of aggregation of these proteins, showed that the dimethylamine and the nitro group on the **B** ring increased inhibition of the *α*-syn aggregation.

The presence of hydroxyl and methoxyl groups was observed in compounds that inhibited the activity of adenosine receptors (A_1,_ A_2A_) and showed antioxidant and anti-inflammatory effects.

In the literature reviewed, only a few studies were found that were also conducted on animal models of PD. The reviewed articles mainly described preliminary studies confirming activity for/against a specific pharmacological target in vitro. In vivo studies were usually conducted as further investigations of the most promising compounds from previous work/s. In the reviewed articles, only five compounds were evaluated in vivo (**7**, **8**, **44**, **46**, and **47**). All studies were conducted in mice. Two different types of animal models of PD were used. Parkinsonism was induced by administration of haloperidol or MPTP.

In the first test, haloperidol-induced cataplexy and motor disturbances such as bradykinesia or rigidity were observed as symptoms of PD. This test is often used as a popular model for the initial assessment of antiparkinsonian activity due to its ease and low cost of use. Furthermore, the results obtained with this model are confirmed in other experimental models of neurodegeneration [88]. In the study used, evaluating the utility of **7** and **8**, haloperidol was administered at a dose of 1 mg/kg for 21 days. Cataplexy was assessed using the cataleptic test (assessment of the cataleptic response on experimental days 7, 14, and 21), the narrow-bar gait test, the open-field test, and the hole-board test. The results showed that, of all the doses tested (10, 20, and 30 mg/kg/day p.o.), the most promising results were obtained for compound **8** at a dose of 30 mg/kg/day [30].

In the second model, administration of the toxin (MPTP) caused motor impairment that resembled the motor disability in PD. Chronic or acute treatment (over 12–24 h) with MPTP damages dopaminergic neurons. This model faithfully reproduced the naturally occurring neurodegeneration in PD [89]. Three compounds **44**, **46**, and **47** were tested in this model [77,82,83]. MPTP was administered acutely at a dose of 20 mg/kg i.p. on the second day (four times every 2 h). After 6 days of MPTP injection, mice showed limb incoordination and motor deficits. These disabilities were assessed using various tests such as the hindlimb test (**44**), the vertical grid test (**44**, **46**, **47**), the rotarod test (**44**), and the coat-hanger test (**46**,**47**). In all cases, the results confirmed an attenuation of motor dysfunction in mice pretreated with the tested compounds at the tested dose (**44**—20 mg/kg/day; **46**—20 mg/kg/day; **47**—30 mg/kg/day; p.o.). Compound **47** also showed improvement in behavioural deficits when administered (at a single dose of 30 mg/kg; p.o.) three days after MPTP injection [83]. Protection of dopaminergic neurons by the tested compounds (**44**, **46**, **47**) against neurotoxin in the MPTP model of PD was also observed by immunohistochemistry studies.”

Taken together, the broad therapeutic possibilities and the ease of modifying the structure of chalcones (both of the **A** and **B** rings) makes these compounds very interesting in the search for new potential drugs for not only PD but many other diseases. In addition, chalcones can be starting structures for synthesis of other heterocyclic compounds with different pharmacological activity.

## Data Availability

Data sharing not applicable.

## Abbreviations

α-syn	alpha-synuclein
ACE	aldoxime chalcone ethers
ACh	acetylcholine
AChE	acetylcholinesterase
AMP	adenosine 5′-monophosphate
AMPK	AMP protein kinase
AR	adenosine receptors
ARE	antioxidant response elements
Aβ	beta-amyloid
A_1_R	adenosine A_1_ receptor
A_1_R^rat^	adenosine A_1_ receptor rat whole brain membranes
A_2A_R	adenosine A_2A_ receptor
A_2A_R^rat^	adenosine A_2A_ receptor rat striatal membranes
BACE-1	β-site amyloid precursor cleaving enzyme 1
BBB	blood-brain barrier
BuChE	butyrylcholinesterase
*ee*BuChE	*equine serum* butyrylcholinesterase
CAT	catalase anti-oxidant markers
ChE	cholinesterase
ChEI	cholinesterase inhibitor
CNS	central nervous system
COMT	catechol-*O*-methyltransferase
COX-2	cyclooxygenase-2
CYP	cytochrome
DA	dopamine
D_1_R	dopamine D_1_ receptors
D_2_R	dopamine D_2_ receptors
ELISA	enzyme-linked immunosorbent assay
*ee*AChE	*electric eel* acetylcholinesterase
eNOS	endothelial NOS
GCase	glucocerebrosidase
GCL	glutamate cysteine ligase
GCLC	glutamate cysteine ligase catalytic subunit
GCLM	glutamate cysteine ligase modifier subunit
GTP	guanosine triphosphate
hMAO A	human monoamine oxidase A
hMAO B	human monoamine oxidase B
HO-1	heme oxygenase-1
H_3_R	histamine H_3_ receptor
iNOS	inducible nitric oxide synthases
IKK	ikappaB kinase
JNK	c-Jun N-terminal kinases
LB	Lewy bodies
LN	Lewy neurons
LPS	lipopolysaccharide
LRRK2	leucine-rich repeat kinase 2
MAO A	monoamine oxidase A
MAO B	monoamine oxidase B
MB-COMT	membrane-bound catechol-*O*-methyltransferase
MDA	malondialdehyde
MG	methylglyoxal
MM/GBSA	molecular mechanics with generalized Born and surface area solvation
MPTP	1-methyl-4-phenyl-1,2,3,6-tetrahydropyridine
rMAO A	rat brain mitochondria MAO A
rMAO B	rat brain mitochondria MAO B
ND	neurodegenerative disorders
NMDA	*N-*methyl-D-aspartate
NMS	non-motor symptoms
nNOS	neuronal nitric oxide synthases
NO	nitric oxide
NOS	nitric oxide synthases
Nrf2	nuclear factor erythroid 2-related factor 2
PAMPA assay	parallel artificial membrane permeability assay
PARP1	poly [ADP-ribose] polymerase 1
PD	Parkinson’s disease
PDE10A	phosphodiesterase 10A
PD-MCI	Parkinson’s disease–mild cognitive impairment
PD-D	Parkinson’s disease dementia
PET	positron emission tomography
p-LIMK1	phosphorylated LIM kinase 1
PSMA1	proteasome 20S subunit alpha 1
PSMB5	proteasome 20S subunit beta 5
PSMB7	proteasome 20S subunit beta 7
PSMB8	proteasome 20S subunit beta 8
ROCK2	pho-associated protein kinase 2
ROS	reactive oxygen species
SN	*substantia nigra*
SOD	superoxide dismutase
ThT	thioflavin T
